# Mucosal Metabolomic Signatures in Chronic Colitis: Novel Insights into the Pathophysiology of Inflammatory Bowel Disease

**DOI:** 10.3390/metabo13070873

**Published:** 2023-07-23

**Authors:** Nathan Calzadilla, Aisha Qazi, Anchal Sharma, Kai Mongan, Shane Comiskey, Jahnavi Manne, Alvin G. Youkhana, Sonam Khanna, Seema Saksena, Pradeep K. Dudeja, Waddah A. Alrefai, Ravinder K. Gill

**Affiliations:** 1 Department of Biomedical Engineering, University of Illinois Chicago, Chicago, IL 60612, USA; ncalza3@uic.edu; 2 Division of Gastroenterology & Hepatology, University of Illinois Chicago, Chicago, IL 60612, USA; aqazi7@uic.edu (A.Q.); anchals@uic.edu (A.S.); kmongan@neomed.edu (K.M.); shanec@uic.edu (S.C.); jmanne2@uic.edu (J.M.); ayoukh4@uic.edu (A.G.Y.); skhann5@uic.edu (S.K.); saksena@uic.edu (S.S.); pkdudeja@uic.edu (P.K.D.); walrefai@uic.edu (W.A.A.); 3 Jesse Brown VA Medical Center, Chicago, IL 60612, USA

**Keywords:** chronic colitis, mucosal inflammation, host–microbe interactions, mucosal metabolomics

## Abstract

Inflammatory bowel diseases (IBD) involve complex interactions among genetic factors, aberrant immune activation, and gut microbial dysbiosis. While metabolomic studies have focused on feces and serum, fewer investigations have examined the intestinal mucosa despite its crucial role in metabolite absorption and transport. The goals of this study were twofold: to test the hypothesis that gut microbial dysbiosis from chronic intestinal inflammation leads to mucosal metabolic alterations suitable for therapeutic targeting, and to address gaps in metabolomic studies of intestinal inflammation that have overlooked the mucosal metabolome. The chronic DSS colitis was induced for five weeks in 7–9-week-old wild-type C57BL/6J male mice followed by microbial profiling with targeted 16srRNA sequencing service. Mucosal metabolite measurements were performed by Metabolon (Morrisville, NC). The data were analyzed using the bioinformatic tools Pathview, MetOrigin, and Metaboanalyst. The novel findings demonstrated increases in several host- and microbe-derived purine, pyrimidine, endocannabinoid, and ceramide metabolites in colitis. Origin analysis revealed that microbial-related tryptophan metabolites kynurenine, anthranilate, 5-hydroxyindoleacetate, and C-glycosyltryptophan were significantly increased in colon mucosa during chronic inflammation and strongly correlated with disease activity. These findings offer new insights into the pathophysiology of IBD and provide novel potential targets for microbial-based therapeutics.

## 1. Introduction

Inflammatory bowel diseases (IBD) are chronic, debilitating illnesses with two major subtypes: ulcerative colitis and Crohn’s disease. Between 1990 and 2017, the number of affected individuals rose from 3.7 million to 6.8 million, with the highest prevalence rates in North America [1]. In addition to diarrhea, abdominal pain, ulcers, and rectal bleeding, IBD carries an increased risk of colon cancer along with several extraintestinal manifestations [2]. Ulcerative colitis is characterized by mucosal inflammation localized to the colon, while Crohn’s disease is characterized by full thickness inflammation anywhere along the gastrointestinal tract with skip lesions [3]. While there are many therapeutic options, treatment is complex, not curative, and carries significant side effects [4]. Moreover, patients with refractory disease often require surgery and possibly parenteral nutrition. Further complicating the clinical picture are the various factors in the pathophysiology of IBD, including host genetic factors, intestinal barrier disruption, immune dysregulation, gut microbial dysbiosis, and altered metabolite pools [5]. This has led to several groups utilizing metabolomic and integrated omic tools to better understand the pathophysiology and progression of IBD [6,7,8,9,10,11,12,13,14,15].

For instance, meta-analysis of gene expression data in IBD patients revealed that ileal and colonic disease have distinct molecular patterns that are not adequately considered with clinical diagnosis and management [16]. These approaches have also been successfully applied to predict patient responses to anti-integrin therapy for the implementation of more efficacious combinatorial treatments [17]. Several studies have also performed microbiota profiling and metabolomics in the serum and feces of IBD patients to detect novel disease signatures [18,19]. Furthermore, human studies provide tremendous value in their translational potential, but due to the myriad of confounding variables in clinical studies, it is much more difficult to reach mechanistic conclusions. Therefore, animal models of IBD are often used to mimic human disease and supplement human studies [20]. Additionally, animal models allow for more uniformity and the ability to capture a disease process at a specific timepoint. For instance, one such study performed untargeted mucosal and serum metabolomics in a model of acute DSS colitis and found that alterations in tryptophan metabolism affected AhR-mediated IL-10 signaling [21]. Moreover, these high throughput approaches have also been used to identify novel drivers of intestinal inflammation such as proteins involved in the actin cytoskeleton and beneficial dietary compounds from human studies with further investigation using animal models to gain mechanistic insight [22,23]. However, there is a need for such studies in additional models that are more reflective of the chronically relapsing and remitting nature of IBD.

Here we performed untargeted colonic mucosal metabolomic profiling in a murine model of chronic DSS colitis that resembles IBD. The objectives of this study were to understand gut microbial changes during chronic colitis, identify differentially altered microbial metabolites, and uncover altered pathways during chronic intestinal inflammation for future study. This study was driven by the hypothesis that chronic colonic inflammation drives unique changes in the mucosal metabolome reflective of IBD due to inflammation-induced gut microbial dysbiosis and disturbed intestinal architecture. 

## 2. Materials and Methods

### 2.1. Animals

All animal studies were performed in accordance with institutional guidelines and regulations and approved by the Animal Care Committees of the University of Illinois at Chicago and the Jesse Brown Veterans Affairs Medical Center (Approval Code: ACC 21-078). Wild-type C57/BL6 mice were purchased from Jackson Laboratory (Bar Harbour, ME, USA). Autoclaved polypropylene cages with corncob bedding were used to house the mice. The mice were provided free access to food and water (Teklad Irradiated LM-485 Mouse/Rat Diet 7912, Envigo, Indianapolis, IN, USA) under a 12 h light/dark cycle. Male mice aged 7–9 weeks were provided either water alone (control group) or water supplemented with 2.5% *w*/*v* dextran sulfate sodium (DSS) (colitis group) for 5 weeks to induce chronic colitis. DSS was administered to mice for three cycles (one week/cycle), alternating with water only between the cycles (DSS was administered during weeks 1, 3, and 5, while water was administered during weeks 2 and 4) [24].

### 2.2. Metabolomic Analysis

Samples were processed and analyzed by Metabolon Inc. (Durham, NC, USA) as previously described [25]. Additional details are provided below. 

#### 2.2.1. Sample Preparation

Hamilton Company’s automated MicroLab STAR^®^ system was used to prepare samples. For quality control purposes, several recovery standards were added prior to the first step in the extraction process. Proteins were precipitated with methanol for 2 min with vigorous shaking, followed by centrifugation for 5 min at 700× *g* to dissociate small molecules bound to protein or trapped in the precipitated protein matrix and recover chemically diverse metabolites (Glen Mills GenoGrinder 2000). The resulting extract was divided into five fractions: two for analysis using two separate reverse phases (RP)/UPLC-MS/MS methods with positive ion mode electrospray ionization (ESI), one for analysis using RP/UPLC-MS/MS with negative ion mode ESI, one for analysis using HILIC/UPLC-MS/MS with negative ion mode ESI, and one for backup. To remove the organic solvent, samples were briefly placed on a TurboVap^®^ (Zymark; Hopkinton, MA, USA). Before preparing for analysis, the sample extracts were dried overnight under nitrogen gas [25].

#### 2.2.2. Ultrahigh Performance Liquid Chromatography–Tandem Mass Spectroscopy (UPLC-MS/MS)

Waters ACQUITY ultra-performance liquid chromatography (UPLC) and a Thermo Scientific Q-Exactive high resolution/accurate mass spectrometer interfaced with a heated electrospray ionization (HESI-II) source and Orbitrap mass analyzer operated at 35,000 mass resolution were used in all methods. The sample extract was dried before being reconstituted in solvents suitable for each of the four methods. To ensure injection and chromatographic consistency, each reconstitution solvent contained a series of standards at fixed concentrations. One aliquot was analyzed under acidic positive ion conditions, which were chromatographically tailored for more hydrophilic substances. In this procedure, the extract was gradient eluted from a C18 column (Waters UPLC BEH C18-2.1 × 100 mm, 1.7 m) using water and methanol containing 0.05% perfluoropentanoic acid (PFPA) and 0.1% formic acid (FA). Another aliquot was examined under acidic positive ion conditions that had been chromatographically tuned for more hydrophobic substances. The extract was gradient-eluted from the same aforementioned C18 column using methanol, acetonitrile, water, 0.05% PFPA, and 0.01% FA and was operated at a higher total organic content in this method. A separate dedicated C18 column was used to evaluate another aliquot under basic negative ion optimized conditions. The basic extracts were gradient-eluted from the column with methanol and water containing 6.5 mM ammonium bicarbonate at pH 8. After being eluted from an HILIC column (Waters UPLC BEH Amide 2.1 × 150 mm, 1.7 m) using a gradient of water and acetonitrile with 10 mM ammonium formate, pH 10.8, the fourth aliquot was subjected to negative ionization analysis. Dynamic exclusion was used to alternate between the MS analysis and data-dependent MSn scans. The scan range varied slightly between methods, but it was between 70 and 1000 *m*/*z*. The raw data files were archived and extracted in the manner described below [25].

#### 2.2.3. Compound Identification and Metabolite Quantification

Using Metabolon’s equipment and software (Durham, NC, USA), raw data were retrieved, peaks were found, and quality control was performed. Compounds were discovered by comparing them to purified standards in the library. The library was built on authenticated standards and included retention time/index (RI), mass-to-charge ratio (*m*/*z*), and chromatographic data (including MS/MS spectrum data). Compound identification was based on the retention index within a limited RI window of the proposed identification, an accurate mass match to the library within 10 ppm, and the MS/MS forward and reverse scores between experimental data and authenticated standards. Peaks were measured using the area-under-the-curve method and normalized to total protein as determined using the Bradford assay [25].

### 2.3. Bioinformatic Analysis

Heatmaps were generated for statistically significant differential metabolites using ComplexHeatMap in R [26,27]. Origin analysis was performed utilizing the online Met-Origin interface [28]. Briefly, a list of significantly altered metabolites with Kyoto Encyclopedia of Genes and Genomes (KEGG) and Human Metabolome Database (HMBD) IDs were uploaded, and we proceeded with simple analysis. Enrichment analysis and sPLSDA analysis were performed using Metaboanalyst [29].

### 2.4. Myeloperoxidase Activity

With some minor modifications, the Krawisz [30] method was used to measure myeloperoxidase activity (MPO) in the distal colon. MPO activity is expressed as units per milligram of tissue after being normalized to the amount of tissue.

### 2.5. Hematoxylin and Eosin Staining 

The intestines were sampled, and they were quickly rinsed in PBS. The tissues were immediately snap-frozen in liquid nitrogen after being embedded in OCT media. Hematoxylin and eosin (H&E) staining was performed according to the manufacturer’s protocol (ScyTek Laboratories, Logan, UT, USA) on longitudinal sections that were 5 µm thick, as previously described [31].

### 2.6. Microbial Sampling and 16S rRNA Sequencing

Cecal contents were collected and immediately snap-frozen in liquid nitrogen. Samples were sent to Zymo Research (Irvine, CA, USA) for 16S rRNA sequencing. Amplicon sequence variant (ASV) analysis was performed with DADA2. Linear discriminant analysis (LDA) was performed with bioBakery [32,33]. 

### 2.7. Statistical Analysis

For metabolite abundances, Welch’s two-sample *t*-test was used to assess the significance and set at a level of *p* < 0.05. A false discovery rate (FDR) method was also applied with a set q < 0.1. Welch’s two-sample *t*-test was used to assess significance for all other comparisons. 

## 3. Results

### 3.1. Gut Microbial Dysbiosis Is Associated with Chronic DSS Treatment

**Figure 1:**
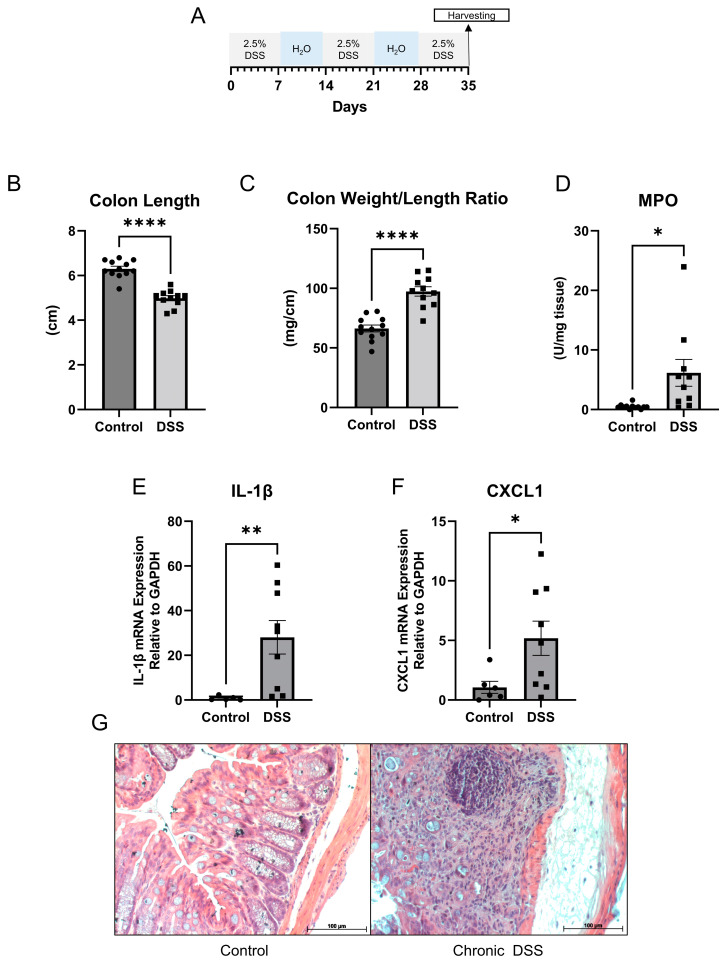
(**A**) Schematic for study design. (**B**) Colon length (cm) (**C**) Colon weight/length ratio (mg/cm) (**D**) Myeloperoxidase (MPO) activity units per mg of tissue (control *n* = 12; dextran sulfate sodium (DSS) *n* = 11). (**E**,**F**) Quantitative PCR for IL-1β and CXCL1 in distal colon mucosa (control *n* = 5; DSS *n* = 9). (**G**) Representative hematoxylin and eosin staining of control and chronic DSS mice. (Welch *t*-test; * *p* < 0.05, ** p < 0.01, **** p < 0.0001).

Mucosal inflammation is a key feature in IBD [34]. Optimal treatment options focusing on complete healing of the mucosa are warranted. To understand the key mucosal alterations occurring during chronic intestinal inflammation, we utilized the chronic DSS colitis to model the relapsing and remitting nature of human IBD. Wild-type mice were administered alternating cycles of 2.5% DSS in drinking water and water alone for five weeks to induce chronic colitis (Figure 1A). We first assessed the severity of experimentally induced inflammation. As shown in Figure 1B and Supplementary Figure S1, colon length was significantly decreased in the group treated with DSS, suggestive of intestinal inflammation. The average colon length decreased from 6.3 cm to 5.0 cm during chronic DSS treatment (*p* < 0.0001). Additionally, the colon weight-to-length ratio, a marker used to determine diarrhea, was significantly increased in the group treated with chronic DSS (*p* < 0.0001) (Figure 1C). Given that another key aspect of colitis is neutrophil infiltration, the severity of inflammation was further determined by measuring myeloperoxidase (MPO) activity in colonic mucosa from the distal colon. Figure 1D showed that the average MPO activity increased from 0.495 units/mg of tissue to 6.163 units/mg of tissue (*p =* 0.0333). Further, qPCR demonstrated increases in the mRNA expression of proinflammatory cytokines IL-1β (*p =* 0.0067) (Figure 1E) and CXCL1 (*p =* 0.0224) (Figure 1F). Finally, representative histological analysis revealed architectural distortion, active inflammation, and a greater than 25% ulcerated area in the distal colon during chronic DSS colitis compared to controls (Figure 1G). 

**Figure 2:**
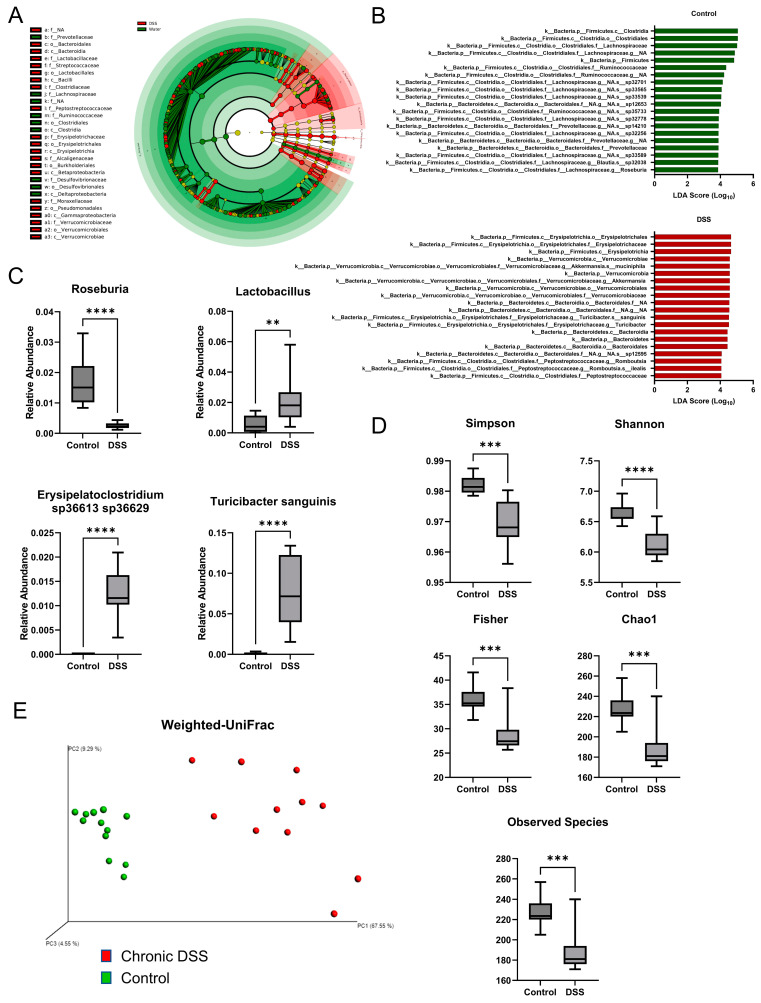
(**A**) Cladogram highlighting taxa associated with control water and dextran sulfate sodium (DSS) treatments based on linear discriminant analysis effect size. (**B**) Top 20 taxa associated with control group based on linear discriminant analysis effect size (LEfSe) analysis (**top**) and top 20 taxa associated with DSS group based on LEfSe analysis (**bottom**). (**C**) Boxplots of relative abundances for various taxa associated with inflammatory bowel diseases (IBD) (*p <* 0.05, Welch *t*-test; control *n* = 12; DSS *n* = 11). (**D**) Boxplots for alpha diversity measures. (**E**) Weighted UniFrac beta diversity plot. (Welch *t*-test; ** *p* < 0.01, *** p < 0.001, **** p < 0.0001).

Gut microbial dysbiosis, another hallmark of chronic colitis, was further examined [35]. 16S rRNA sequencing was performed on the cecal contents, followed by LEfSe analysis to identify differentially altered microbes. The cladogram depicted in Figure 2A shows the various taxonomic levels of microbes associated with chronic colitis and control groups and their taxonomic relationship to each other from LEfSe (linear discriminant analysis effect size) analysis. A tabular summary of these taxa are depicted with their effect size and *p*-value in Supplemental Table S1. Additionally, changes observed in this study that are consistent with IBD are depicted in Supplemental Table S2 [36,37,38,39,40,41,42]. Figure 2B depicts the top 20 taxa associated with the control and DSS groups, and Supplemental Figures S2 and S3 depict all taxa. Of note, the phyla Verrucomicrobia, Bacteroidetes, and Actinobacteria were significantly associated with chronic DSS colitis. Furthermore, alpha diversity was assessed using several indices, including the total number of observed species, Chao1, Fisher, Shannon Diversity, and Simpson index. Overall, the data demonstrated a loss of alpha diversity within samples of mice with chronic colitis (Figure 2D). Specifically, the overall species observed dropped from 226.7 in the control group to 189.5 in the colitis group (*p* = 0.0001). Additionally, the Chao1, Fisher, Simpson, and Shannon diversity indices, which consider both the number of species and the abundance of each species, decreased from 226.8 in the control group to 189.5 during chronic colitis (*p* = 0.0001), 35.84 to 29.03 (*p =* 0.0001), 0.9819 to 0.9696 (*p* = 0.0002), and 6.629 to 6.117 (*p* < 0.0001), respectively. As expected, chronic colitis led to both a decrease in the overall number of species and the distribution of species. Furthermore, beta diversity was assessed using the weighted UniFrac method [43], which accounts for the presence of organisms, their abundance, and their taxonomic relationship to each other. The analysis showed that each sample’s microbial communities clustered according to their treatment group (Figure 2E). Furthermore, a hallmark of microbial changes in IBD is a loss of short-chain fatty acid-producing bacteria [44]. There was a decrease in the relative abundance of the genus *Roseburia* (*p* < 0.001) (Figure 2C) and several *Roseburia* species (Supplemental Figure S4), which were identified as biomarkers for the control groups through LEfSe analysis. There have also been reported increases in the genus Lactobacillus in IBD cohorts [37], which were also found in the chronic DSS samples (Figure 2C, *p =* 0.0070). Further, we found increases in *Turicibacter sanguinis* (*p* < 0.001) and *Erysipelatoclostridium* species (*p* < 0.001), which also correspond well with previous reports on human cohorts with IBD (Figure 2C) [36]. Overall, our findings showed that chronic DSS treatment in mice resulted in chronic colitis phenotype with associated dysbiosis, providing an adequate model to further explore the mucosal changes that occur in the colon during on-again, off-again inflammatory insults.

### 3.2. Chronic Colitis Leads to Distinct Metabolomic Profile in the Colonic Mucosa

**Figure 3:**
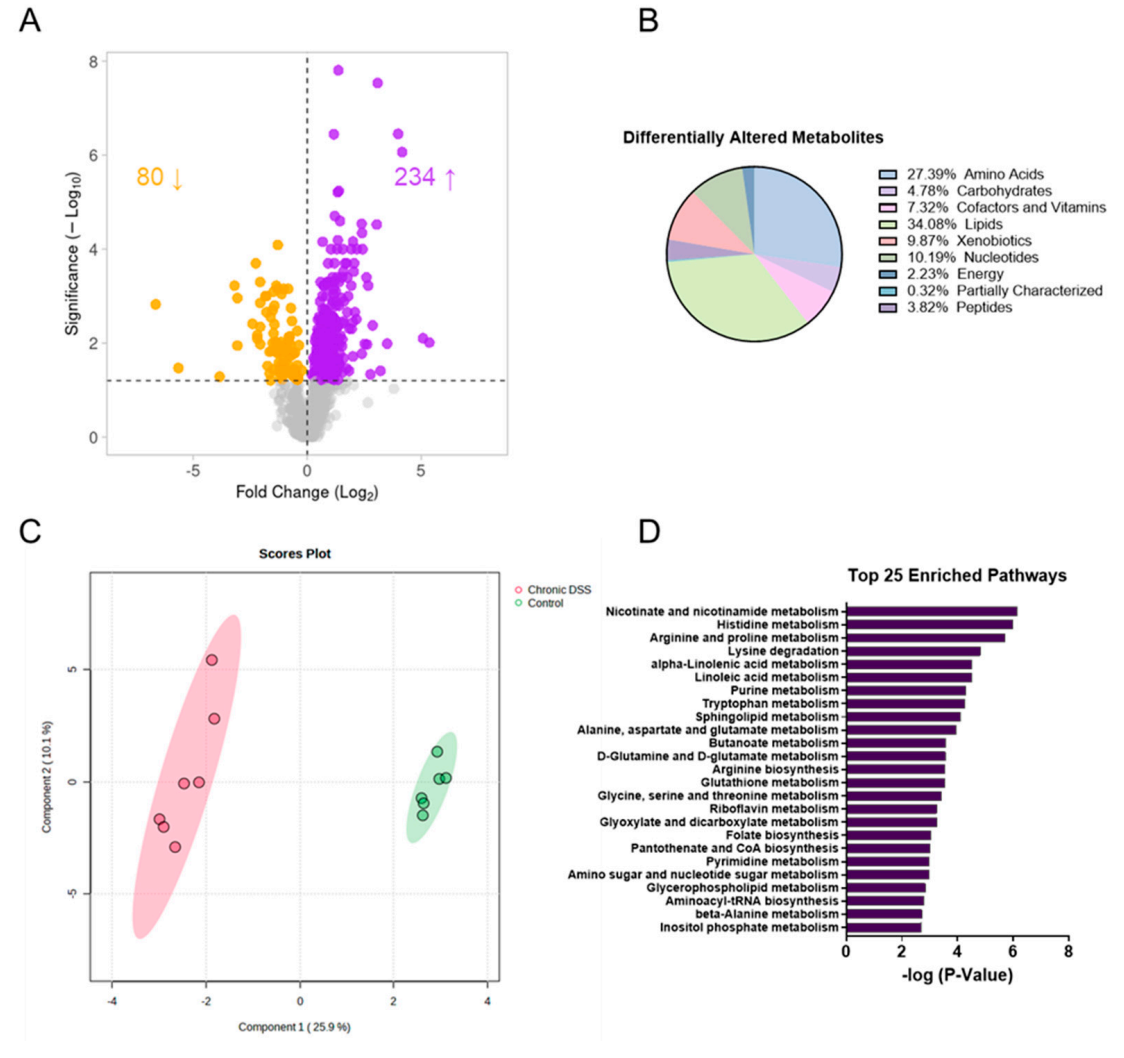
(**A**) Volcano plot (the log2 of the fold change of metabolites in wild-type (WT) dextran sulfate sodium (DSS) compared to WT was plotted against the −log10 of the *p*-value) where the up-facing arrow indicates the number of significantly metabolites increased in abundance and the down-facing arrow indicates the number of decreased and (**B**) pie chart showing the proportion of differentially altered metabolites by metabolite subtype. (**C**) Sparse partial least squares discriminant analysis (sPLSDA) plots generated in Metaboanalyst using the Statistical Analysis function. (**D**) Top 25 enriched pathways in mucosa of control vs. DSS treated mice.

Given that functional changes in the gut microbiome are manifested through metabolic alterations, untargeted metabolomic analysis was next performed to take an unbiased approach to discovering novel changes in the intestinal mucosa during chronic inflammation. The analysis revealed a unique metabolic signature in the colon mucosa after chronic colitis. Overall, there were 314 differentially altered metabolites (*p* < 0.05, q < 0.1), with 80 being decreased and 234 metabolites increased in the colonic mucosa of mice with chronic colitis (Figure 3A, Supplemental Figure S5). Of the 314, the largest proportion of altered metabolites was involved in lipid metabolism, with the next largest group involved in amino acid metabolism. The same trend was observed for both the increased and decreased metabolites when compared to the control (Figure 3B). Additionally, sparse partial least squares discriminant analysis (sPLSDA) revealed two distinct clusters when comparing the colon mucosa metabolome of control mice to those with chronic colitis (Figure 3C). This assessment was followed up using enrichment analysis, which considers how many metabolites are altered within a given Kyoto Encyclopedia of Genes and Genomes (KEGG) pathway (Figure 3D). Of note, several nucleotide and amino acid metabolism pathways were among the most enriched.

### 3.3. Chronic Colitis Leads to Increased Mucosal Localization of Inflammatory Metabolites and Impaired Enterohepatic Circulation

**Figure 4:**
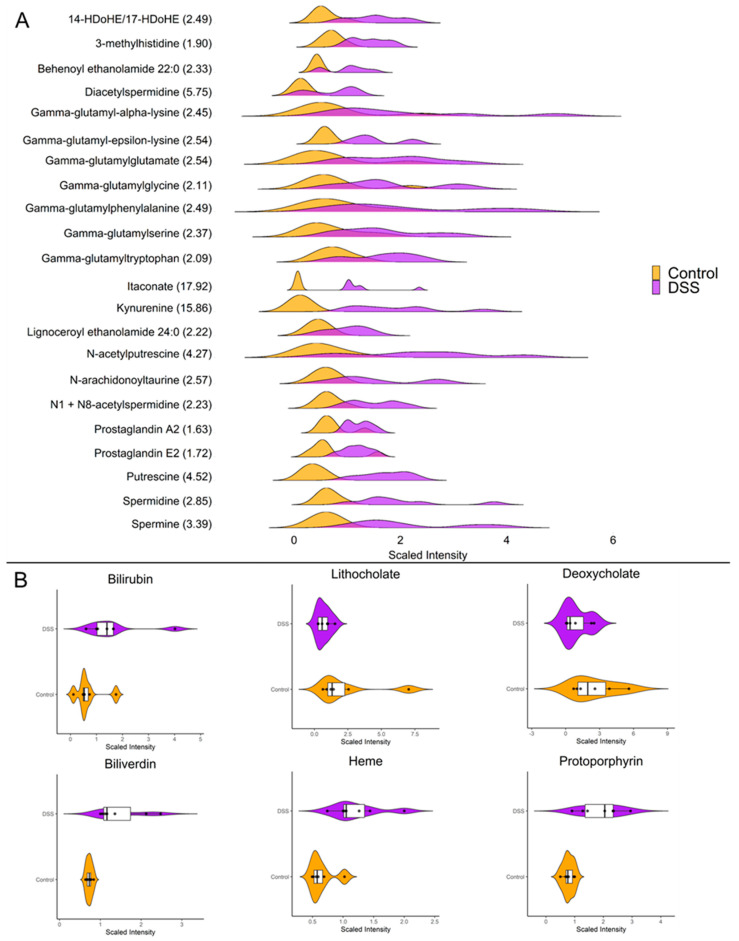
(**A**) Ridgeline plot showing the distributions of differentially altered metabolites with known roles as inflammatory mediators (*p <* 0.05, q < 0.01, Welch *t*-test; control *n* = 6; DSS *n* = 7). The values within parenthesis indicate the fold change between DSS and control. (**B**) Raincloud plots showing abundance and distributions of differentially altered enterohepatic metabolites (*p* < 0.05, q < 0.01, Welch *t*-test; control *n* = 6; DSS *n* = 7).

Our investigations revealed striking differences in the mucosal abundances of several metabolites that are implicated in inflammatory processes such as polyamines, amino acid metabolites, eicosanoids, and TCA cycle intermediates. Figure 4A highlights the distributions of the significantly altered inflammatory mediators in the mucosa during chronic DSS colitis. It is worth highlighting the vast increase in mucosal itaconate levels of nearly 18-fold (*p* < 0.001) during chronic inflammation, as it has been shown to have anti-inflammatory properties in models for sepsis, autoimmune disease, and reperfusion injury [45]. Furthermore, kynurenine, a tryptophan metabolite known to be upregulated during inflammation, was increased nearly 16-fold (*p* < 0.001) in the mucosa after chronic DSS treatment. Additionally, our data revealed several interesting changes in metabolites involved in enterohepatic circulation (Figure 4B). For instance, the secondary bile acids deoxycholate and lithocholate, which are directly influenced by gut microbiota, are significantly decreased during chronic colitis (*p* < 0.05). Moreover, heme and its degradation products bilirubin, biliverdin, and protoporphyrin IX were significantly increased in the chronically inflamed mucosa, suggesting increased synthesis and/or absorption of erythrocyte byproducts. 

### 3.4. Contribution of Microbial Mucosal Metabolomic Profile during Chronic Colitis

**Figure 5:**
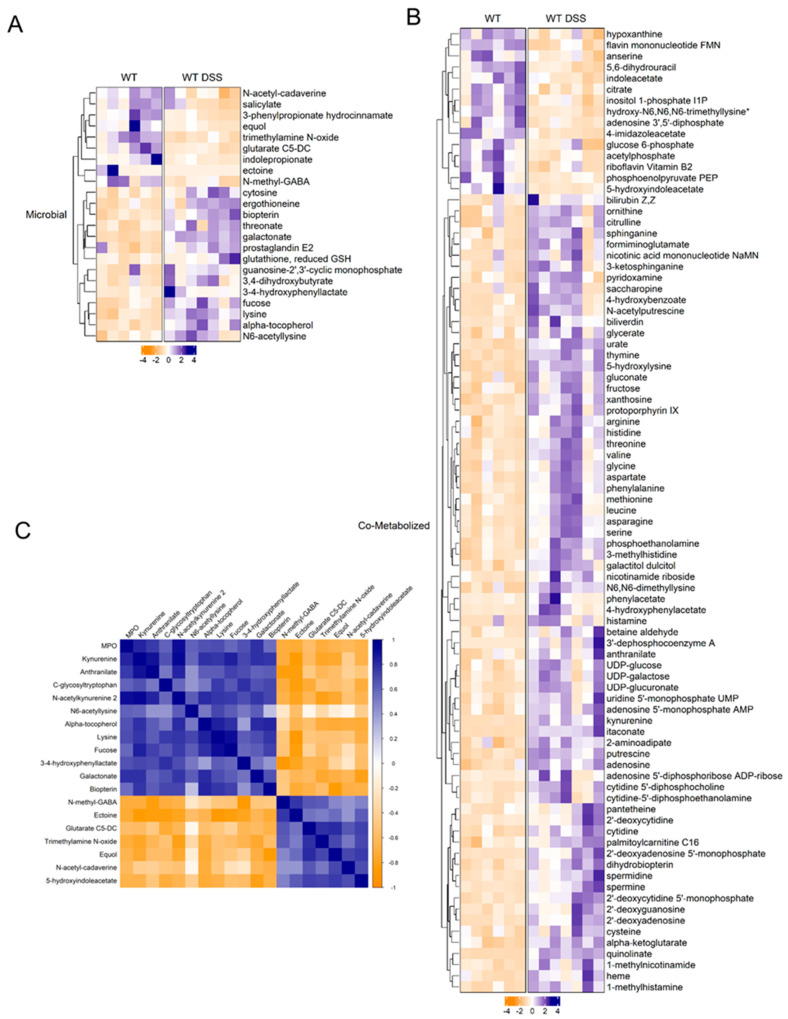
(**A**) Heatmap of microbial-related and (**B**) co-metabolized differentially altered metabolites between control and chronic colitis mice (*p* < 0.05, q < 0.1, Welch *t*-test; control *n* = 6; DSS *n* = 7. (**C**) Correlation plot showing Spearman correlation coefficients between microbial metabolites and MPO activity that were statistically significant (*p <* 0.05). * indicates compounds with lack of reference standard acquisition but their identity is based upon orthogonal information.

An unbiased approach was utilized to examine the potential link between gut microbial dysbiosis and the altered metabolic profile in the colonic mucosa. The bioinformatic tool Met-Origin was utilized to aid in classifying metabolites as related to microbes or co-metabolized by both the host and microbes. This analysis revealed a multitude of metabolites that were altered, at least in part, through changes in gut microbial composition (Figure 5). Our analysis showed 23 microbial-related metabolites differentially altered in chronic colitis (Figure 5A). Interestingly, fucose was increased in the colon mucosa of mice with chronic colitis. Additionally, several differentially altered metabolites were significantly associated with disease severity assessed by MPO levels (Figure 5B, Supplemental Figure S6). Our analysis revealed that in addition to several co-metabolized nucleotides highlighted in Figure 5, there were increases in heme-related metabolites protoporphyrin IX and biliverdin in the colonic mucosa of mice with chronic colitis (Figure 5C) that are also highlighted in Figure 4B in more detail. This may suggest increased heme breakdown and compensatory synthesis in response to chronic intestinal inflammation. Ultimately, this analysis allows for a better understanding of the contribution of gut microbial dysbiosis to the metabolomic alterations in the mucosa of mice with chronic colitis. 

### 3.5. Chronic Colitis Alters Mucosal Dietary, Nucleotide, Amino Acid, and Lipid Metabolism

**Figure 6:**
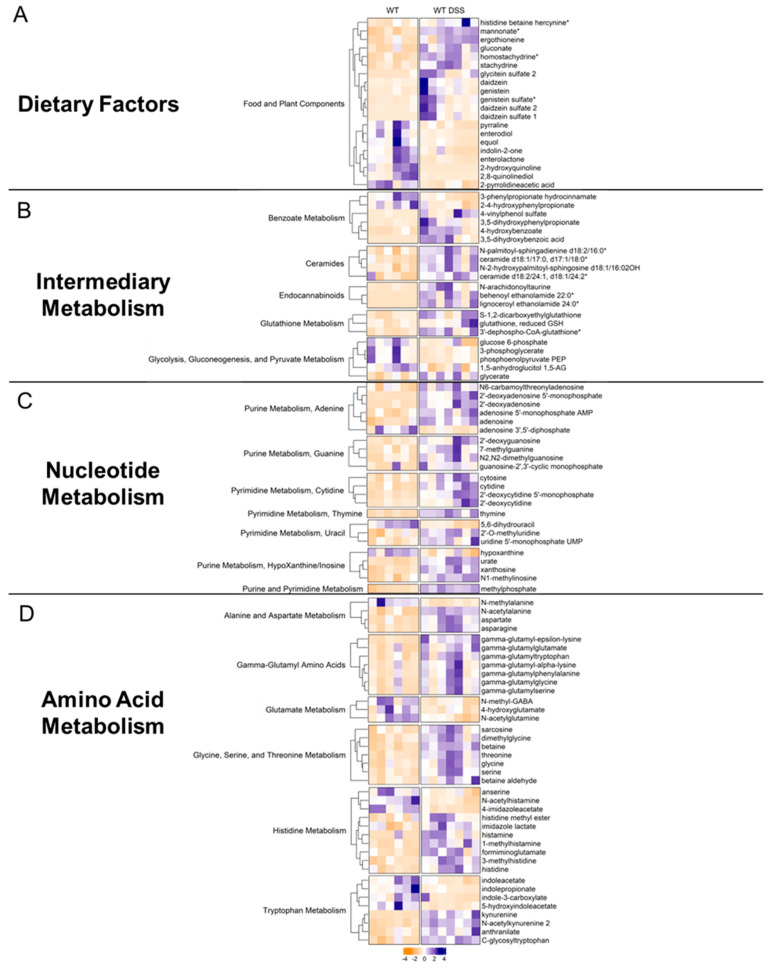
Heatmaps of differentially altered metabolites (*p* < 0.05, q < 0.1, Welch *t*-test) in (**A**) dietary factors, (**B**) intermediary metabolism, (**C**) nucleotide metabolism, and (**D**) amino acid metabolism. Heatmap was scaled from −4 to 4 (control = 6; DSS *n* = 7). * indicates compounds with lack of reference standard acquisition but their identity is based upon orthogonal information.

Food and plant components: Differentially altered metabolites were subsequently organized by their metabolic pathway. For instance, there were several alterations in dietary and plant-derived metabolites in the colonic mucosa of mice with chronic colitis (Figure 6A). Stachydrine and ergothioneine, for instance, are both increased in the mucosa during chronic colitis. Given that these are dietary metabolites, our findings suggest altered colonic nutrient absorption in response to inflammation. Our analysis also revealed alterations in several nucleotide metabolism pathways. 

Intermediary metabolism: Several interesting increases in other classes of metabolites were also found. For instance, endocannabinoids (Figure 6B), a class of compounds newly emerging as a possible therapeutic target in IBD, showed general increases in the mucosa of mice with chronic colitis. Other interesting findings included increases in ceramides. Furthermore, the mucosa of mice with chronic colitis demonstrated decreased glycolytic intermediates such as glucose 6-phosphate, phosphoenolpyruvate, and 3-phosphoglycerate, suggesting decreased glycolytic activity (Supplemental Figure S7).

Nucleotide metabolism: Generally, there were increases in metabolites involved in adenine, guanine, cytidine, thymine, uracil, and inosine metabolism (Figure 6C, Supplemental Figure S8) in the colon mucosa of chronic colitis mice, suggesting increased nucleotide production due to increased epithelial cell turnover. For instance, adenosine was increased in the mucosa during chronic colitis. Interestingly, adenosine receptor agonists have been shown to mitigate the effects of DSS-induced colitis through NF-κB signaling. Another interesting finding was the increase in methylated nucleotide metabolites such as N1-methylinosine, 2′-O-methyluridine, and N2, N2-dimethylguanosine, suggesting changes in genetic regulation. 

Amino acid metabolism: Our data showed that gamma-glutamyl amino acids were increased during chronic colitis (Figure 6D, Supplemental Figure S9). Interestingly, decreases in microbial indole tryptophan metabolites and increases in co-metabolized kynurenine were also found, revealing alterations in tryptophan metabolism within intestinal mucosa during inflammation. Increases in the amino acids glycine, serine, threonine, histidine, aspartate, and asparagine were also identified, suggesting altered transport and/or metabolism in the intestinal mucosa during chronic inflammation. This analysis revealed several findings corroborated in human cohorts of IBD and other IBD models while also describing novel metabolomic findings in the colonic mucosa during chronic colitis. 

## 4. Discussion

In this study, it was hypothesized that chronic colonic inflammation results in a unique microbially influenced mucosal metabolomic profile, providing new insight into the pathophysiology of inflammatory bowel diseases (IBD). This study investigated the gut microbial composition and mucosal metabolome of mice with induced chronic colitis. This work found that chronic colitis resulted in increased myeloperoxidase activity in the colon, as well as increased levels of proinflammatory cytokines IL-1β and CXCL1. Gut microbial analysis revealed expected losses in alpha diversity, two distinct beta diversity clusters, and microbial composition changes consistent with IBD patients. Furthermore, mucosal metabolomics revealed 328 differentially altered metabolites with several enriched metabolic pathways. These changes were alterations in microbial-related metabolites, food and plant components, nucleotides, amino acids, and lipids. 

### 4.1. Facts and Perspectives

Gut microbial dysbiosis is a critical factor in the pathophysiology of IBD [35]. In this regard, several groups have investigated changes in the gut microbiome in IBD patients and in animal models for IBD with one characteristic feature being a loss of alpha diversity [46,47], a measure for the total number of species as well as the abundance of such species, which were observed in the present study. Additionally, beta diversity, which has also been consistently affected by IBD [47], was also found to be affected by induced chronic colitis in the current study. Furthermore, several characteristic microbial changes have been highlighted in the chosen disease model that have also been reported in IBD patients. The genus Roseburia was identified as a characteristic feature of untreated control mice and was significantly decreased in abundance in mice with chronic colitis. Roseburia are known to produce short-chain fatty acids, which have been shown to decrease in abundance in IBD, and administration of Roseburia intestinalis supernatant has been shown to alleviate induce colitis [48,49,50]. Although there was not a statistically significant decrease in the mucosal levels of the SCFA butyrate, there was a trend towards a decrease in the chronically inflamed mucosa. This could be due to SCFAs serving as the key fuel for colonocytes and thus being rapidly metabolized. There was also an increase in the genus Lactobacillus, Turicibacter sanguinis, and an Erysipleatoclostridium species, which have also been observed in IBD patients [36,37,39]. 

While it was not surprising to observe distinct mucosal metabolic profiles during chronic colitis, there were several findings that have been corroborated in IBD patients parallel to novel discoveries that can be used for further mechanistic inquiries. For example, decreases in indole metabolites such as indoleacetate, indolepropionate (IPA), and indole-3-carboxylate have been shown in the serum of subjects with ulcerative colitis with IPA serving as a marker for disease remission [51]. Interestingly, our data show that the decreases in these indole metabolites are occuring in the intestinal mucosa during inflammation. This suggests that the decreases in the serum of those with IBD is due to decreased microbial production and not increased transport in mucosal epithelial or immune cells. Collectively, these findings can be exploited for the benefit of patients with IBD through microbial-based therapy with cocktails of microbes capable of synthesizing beneficial indole metabolites from tryptophan. Regarding dietary factors, increases in ergothioneine and stachydrine were found in the inflamed mucosa. Stachydrine has been shown to have anti-inflammatory properties in primary human chondrocytes isolated from subjects with osteoarthritis that were treated with IL-1β [52]. Ergothioneine was previously shown to also demonstrate protective effects against DSS-induced colitis by decreasing MPO activity levels and improving intestinal barrier function [53]. Taken together, our findings suggest a compensatory increase in the absorption of anti-inflammatory metabolites stachydrine and ergothioneine in response to mucosal inflammation. This suggests that increasing epithelial absorption of stachydrine and ergothioneine could be used as a potential therapeutic approach in IBD, possibly by increasing the activity of the ergothioneine transporter [54]. Increases in mucosal ceramides, which have been associated with increased inflammation in the mucosa of subjects with IBD, were found [55]. It was also found that the microbial-derived metabolite fucose, a sugar known to participate in the formation of Lewis antigens, was increased in the colon mucosa of mice with chronic colitis. This is particularly notable, as Lewis antigens have been associated with ulcerative colitis [56]. In fact, IgG and IgM reactivity has been shown to be increased towards fucosylated oligosaccharides in the serum of patients with Crohn’s disease, implicating fucose-carrying Bacteroides species in intestinal inflammation [57]. In fact, our analysis identified the order Bacteroidales as a key distinguishing feature for mice with chronic colitis. Considering these findings, therapeutics targeting fucose degradation could serve as a novel strategy for inflammation in IBD. 

The present study also identified several altered metabolic pathways in response to chronic colitis. There were decreased levels of glycolytic intermediates, which is important to note, as a previous study found that patients with IBD had antibodies that recognized several glycolytic enzymes as antigens [58]. One such enzyme was phosphoglycerate mutase, the enzyme responsible for conversion of glycerate-3P to glycerate-2P. Our rendering of the KEGG glycolysis pathway demonstrated decreased levels of glycerate-2P in the intestinal mucosa during chronic colitis, highlighting the need to further explore the implications of disrupted glycolysis. Our data also highlight novel increases in endocannabinoids in the colon mucosa during chronic inflammation. This is an important finding, as the role of the endocannabinoid system in IBD is an emerging area of interest [59]. Moreover, this work found increases in several ceramides, a class of sphingolipids, which are emerging as a new area of investigation in IBD [55]. Ultimately, this study provides a comprehensive report of alterations in mucosal metabolism during chronic inflammation in a relevant model that highlights microbial contributions, novel pathways for future investigation, and identifies newer therapeutic targets for intestinal inflammation. 

This work characterized the gut microbial alterations associated with chronic inflammation and further elucidated the impacts of those changes by analyzing differentially altered microbial-related metabolites. For instance, these studies revealed increases in heme degradation products protoporphyrin IX and biliverdin within the chronically inflamed colon mucosa. Simultaneously, we found that *Turicibacter sanguinis*, a bacterial species that synthesizes biliverdin through heme oxygenase [60], and the family of bacteria *Erysipelotrichaceae,* which are known to utilize protoporphyrin IX as a substrate [61], were both increased in the intestinal lumens of mice with chronic colitis. Our study yielded significant findings indicating elevated levels of various purine, pyrimidine, endocannabinoid, and ceramide metabolites derived from both the host and the gut microbiota in colitis with the potential to both serve as disease biomarkers and as areas of future mechanistic investigation. Notably, our analysis of the origin of these metabolites revealed a microbial contribution, particularly in the case of tryptophan metabolites, including kynurenine, anthranilate, 5-hydroxyindoleacetate, and C-glycosyltryptophan. These microbial-related tryptophan metabolites displayed substantial increases within the colon mucosa during chronic inflammation and exhibited strong correlations with disease activity. Consequently, these findings provide opportunity to develop targeted therapies for harnessing the beneficial metabolites through engineered microbes that can break the proinflammatory metabolites or shunt them towards the synthesis of beneficial compounds. For example, kynurenine aminotransferase-expressing microbes can be utilized to convert increased kynurenine into kynurenate [62]. These findings provide valuable insights into the underlying pathophysiology of IBD and present promising new targets for the development of microbial-based therapeutics.

### 4.2. Strengths of the Study

This study utilized a preclinical translational model for IBD in the form of chronic DSS colitis that mimics remitting and relapsing inflammation observed in human disease. Furthermore, while several metabolomic studies in IBD have focused on less invasive biological compartments such as serum and feces, the present study rigorously examined the mucosal metabolome—an understudied tissue compartment. Moreover, this work utilized a discovery approach with the integration of gut microbiota and mucosal metabolites. This resulted in the identification of differentially altered microbial metabolites resulting from gut microbial dysbiosis that can be utilized as potential biomarkers and targeted with microbial-based therapeutics. The limitations of our study include that metabolomic profiling and microbial sequencing were performed on two distinct sets of mice. However, the approaches utilized in this work were able to identify microbial contributions to microbial-related metabolic changes with MetOrigin. Furthermore, our study used only male mice and should be repeated in female mice at various stages of their estrous cycles, with those stages taken under consideration as a variable. While there are not strong differences in the incidence of IBD between males and females, there are some slight differences that require future study [63]. For instance, females tend to have a higher risk of CD while males tend to have a higher risk of UC. Future studies can focus on further exploring the mechanistic processes involved in the disruption of identified metabolic pathways by separating the mucosa into epithelial cell fractions and lamina propria fractions to better understand which changes are coming from epithelial cells or immune cells specifically. 

## 5. Conclusions

In summary, our studies provide a comprehensive view into the metabolomic alterations in the intestinal mucosa during chronic inflammation that adds to the growing number of studies utilizing metabolomics to gain new insight into disease [64,65]. This study identified novel mucosal metabolites that could be used as potential inflammatory biomarkers and that should warrant further investigation such as disturbances in glycolytic metabolism, endocannabinoids, and ceramides. Finally, this work identified several potential therapeutic targets: increasing microbial production of indole metabolites, shunting kynurenine pathway, targeting fucose degradation, and increasing epithelial cell ergothioneine and stachydrine influx. 

## Data Availability

All data is publicly available as part of supplemental data.

## Supplementary Materials

The following supporting information can be downloaded at: https://www.mdpi.com/article/10.3390/metabo13070873/s1, Figure S1: Representative colon pictures; Figure S2: LEfSe analysis of taxa for control mice; Figure S3: LEfSe analysis of taxa for chronic DSS mice; Figure S4: Relative abundance of *Roseburia* species; Figure S5: Differentially altered mucosal metabolites; Figure S6: Associations between microbial metabolites and MPO; Figure S7: Metabolic alterations in glycolysis/gluconeogenesis; Figure S8: Metabolic alterations in pyrimidine and purine metabolism; Figure S9: Metabolic alterations in amino acid metabolism; Table S1: Notable changes in gut microbiota determine by LEfSe; Table S2: Overlapping microbiota changes with human IBD; Data S1: Gut microbial composition across samples; Data S2: Colon mucosa metabolomics.

## References

[1] Kaplan G.G., Ng S.C. (2017). Understanding and Preventing the Global Increase of Inflammatory Bowel Disease. Gastroenterology.

[2] Guan Q. (2019). A Comprehensive Review and Update on the Pathogenesis of Inflammatory Bowel Disease. J. Immunol. Res..

[3] Gohil K., Carramusa B. (2014). Ulcerative Colitis and Crohn’s Disease. P T.

[4] Al-Bawardy B., Shivashankar R., Proctor D.D. (2021). Novel and Emerging Therapies for Inflammatory Bowel Disease. Front. Pharmacol..

[5] Chang J.T. (2020). Pathophysiology of Inflammatory Bowel Diseases. N. Engl. J. Med..

[6] Lloyd-Price J., Arze C., Ananthakrishnan A.N., Schirmer M., Avila-Pacheco J., Poon T.W., Andrews E., Ajami N.J., Bonham K.S., Brislawn C.J. (2019). Multi-Omics of the Gut Microbial Ecosystem in Inflammatory Bowel Diseases. Nature.

[7] Franzosa E.A., Sirota-Madi A., Avila-Pacheco J., Fornelos N., Haiser H.J., Reinker S., Vatanen T., Hall A.B., Mallick H., McIver L.J. (2019). Gut Microbiome Structure and Metabolic Activity in Inflammatory Bowel Disease. Nat. Microbiol..

[8] Zheng L., Wen X.-L., Duan S.-L. (2022). Role of Metabolites Derived from Gut Microbiota in Inflammatory Bowel Disease. World J. Clin. Cases.

[9] Aldars-García L., Gisbert J.P., Chaparro M. (2021). Metabolomics Insights into Inflammatory Bowel Disease: A Comprehensive Review. Pharmaceuticals.

[10] Kolho K.-L., Pessia A., Jaakkola T., de Vos W.M., Velagapudi V. (2017). Faecal and Serum Metabolomics in Paediatric Inflammatory Bowel Disease. J. Crohn’s Colitis.

[11] Le Gall G., Noor S.O., Ridgway K., Scovell L., Jamieson C., Johnson I.T., Colquhoun I.J., Kemsley E.K., Narbad A. (2011). Metabolomics of Fecal Extracts Detects Altered Metabolic Activity of Gut Microbiota in Ulcerative Colitis and Irritable Bowel Syndrome. J. Proteome Res..

[12] Bjerrum J.T., Wang Y., Hao F., Coskun M., Ludwig C., Günther U., Nielsen O.H. (2015). Metabonomics of Human Fecal Extracts Characterize Ulcerative Colitis, Crohn’s Disease and Healthy Individuals. Metabolomics.

[13] Manzella C.R., Jayawardena D., Pagani W., Li Y., Alrefai W.A., Bauer J., Jung B., Weber C.R., Gill R.K. (2020). Serum Serotonin Differentiates Between Disease Activity States in Crohn’s Patients. Inflamm. Bowel Dis..

[14] De Preter V., Machiels K., Joossens M., Arijs I., Matthys C., Vermeire S., Rutgeerts P., Verbeke K. (2015). Faecal Metabolite Profiling Identifies Medium-Chain Fatty Acids as Discriminating Compounds in IBD. Gut.

[15] Jansson J., Willing B., Lucio M., Fekete A., Dicksved J., Halfvarson J., Tysk C., Schmitt-Kopplin P. (2009). Metabolomics Reveals Metabolic Biomarkers of Crohn’s Disease. PLoS ONE.

[16] Perez K., Ngollo M., Rabinowitz K., Hammoudi N., Seksik P., Xavier R.J., Daly M.J., Dotan I., Le Bourhis L., Allez M. (2022). Meta-Analysis of IBD Gut Samples Gene Expression Identifies Specific Markers of Ileal and Colonic Diseases. Inflamm. Bowel Dis..

[17] Abreu M.T., Davies J.M., Quintero M.A., Delmas A., Diaz S., Martinez C.D., Venables T., Reich A., Crynen G., Deshpande A.R. (2022). Transcriptional Behavior of Regulatory T Cells Predicts IBD Patient Responses to Vedolizumab Therapy. Inflamm. Bowel Dis..

[18] Gallagher K., Catesson A., Griffin J.L., Holmes E., Williams H.R.T. (2021). Metabolomic Analysis in Inflammatory Bowel Disease: A Systematic Review. J. Crohn’s Colitis.

[19] Aldars-García L., Chaparro M., Gisbert J.P. (2021). Systematic Review: The Gut Microbiome and Its Potential Clinical Application in Inflammatory Bowel Disease. Microorganisms.

[20] Baydi Z., Limami Y., Khalki L., Zaid N., Naya A., Mtairag E.M., Oudghiri M., Zaid Y. (2021). An Update of Research Animal Models of Inflammatory Bowel Disease. Sci. World J..

[21] Lanis J.M., Alexeev E.E., Curtis V.F., Kitzenberg D.A., Kao D.J., Battista K.D., Gerich M.E., Glover L.E., Kominsky D.J., Colgan S.P. (2017). Tryptophan Metabolite Activation of the Aryl Hydrocarbon Receptor Regulates IL-10 Receptor Expression on Intestinal Epithelia. Mucosal. Immunol..

[22] Chen Y., Mai Q., Chen Z., Lin T., Cai Y., Han J., Wang Y., Zhang M., Tan S., Wu Z. (2023). Dietary Palmitoleic Acid Reprograms Gut Microbiota and Improves Biological Therapy against Colitis. Gut Microbes.

[23] Kang S., Kim J., Park A., Koh M., Shin W., Park G., Lee T.A., Kim H.J., Han H., Kim Y. (2023). TRIM40 Is a Pathogenic Driver of Inflammatory Bowel Disease Subverting Intestinal Barrier Integrity. Nat. Commun..

[24] Chassaing B., Aitken J.D., Malleshappa M., Vijay-Kumar M. (2014). Dextran Sulfate Sodium (DSS)-Induced Colitis in Mice. Curr. Protoc. Immunol..

[25] Guo L., Milburn M.V., Ryals J.A., Lonergan S.C., Mitchell M.W., Wulff J.E., Alexander D.C., Evans A.M., Bridgewater B., Miller L. (2015). Plasma Metabolomic Profiles Enhance Precision Medicine for Volunteers of Normal Health. Proc. Natl. Acad. Sci. USA.

[26] Gu Z. (2022). Complex Heatmap Visualization. iMeta.

[27] Gu Z., Eils R., Schlesner M. (2016). Complex Heatmaps Reveal Patterns and Correlations in Multidimensional Genomic Data. Bioinformatics.

[28] Yu G., Xu C., Zhang D., Ju F., Ni Y. (2022). MetOrigin: Discriminating the Origins of Microbial Metabolites for Integrative Analysis of the Gut Microbiome and Metabolome. iMeta.

[29] Xia J., Psychogios N., Young N., Wishart D.S. (2009). MetaboAnalyst: A Web Server for Metabolomic Data Analysis and Interpretation. Nucleic Acids Res..

[30] Krawisz J.E., Sharon P., Stenson W.F. (1984). Quantitative Assay for Acute Intestinal Inflammation Based on Myeloperoxidase Activity. Assessment of Inflammation in Rat and Hamster Models. Gastroenterology.

[31] Kumar A., Anbazhagan A.N., Coffing H., Chatterjee I., Priyamvada S., Gujral T., Saksena S., Gill R.K., Alrefai W.A., Borthakur A. (2016). Lactobacillus Acidophilus Counteracts Inhibition of NHE3 and DRA Expression and Alleviates Diarrheal Phenotype in Mice Infected with Citrobacter Rodentium. Am. J. Physiol. Gastrointest. Liver Physiol..

[32] McIver L.J., Abu-Ali G., Franzosa E.A., Schwager R., Morgan X.C., Waldron L., Segata N., Huttenhower C. (2018). BioBakery: A Meta’omic Analysis Environment. Bioinformatics.

[33] Beghini F., McIver L.J., Blanco-Míguez A., Dubois L., Asnicar F., Maharjan S., Mailyan A., Manghi P., Scholz M., Thomas A.M. (2021). Integrating Taxonomic, Functional, and Strain-Level Profiling of Diverse Microbial Communities with BioBakery 3. eLife.

[34] Ahmad R., Sorrell M.F., Batra S.K., Dhawan P., Singh A.B. (2017). Gut Permeability and Mucosal Inflammation: Bad, Good or Context Dependent. Mucosal. Immunol..

[35] Santana P.T., Rosas S.L.B., Ribeiro B.E., Marinho Y., de Souza H.S.P. (2022). Dysbiosis in Inflammatory Bowel Disease: Pathogenic Role and Potential Therapeutic Targets. Int. J. Mol. Sci..

[36] Qiu Z., Yang H., Rong L., Ding W., Chen J., Zhong L. (2017). Targeted Metagenome Based Analyses Show Gut Microbial Diversity of Inflammatory Bowel Disease Patients. Indian J. Microbiol..

[37] Wang W., Chen L., Zhou R., Wang X., Song L., Huang S., Wang G., Xia B. (2014). Increased Proportions of Bifidobacterium and the Lactobacillus Group and Loss of Butyrate-Producing Bacteria in Inflammatory Bowel Disease. J. Clin. Microbiol..

[38] Glover J.S., Browning B.D., Ticer T.D., Engevik A.C., Engevik M.A. (2022). Acinetobacter Calcoaceticus Is Well Adapted to Withstand Intestinal Stressors and Modulate the Gut Epithelium. Front. Physiol..

[39] Facchin S., Vitulo N., Calgaro M., Buda A., Romualdi C., Pohl D., Perini B., Lorenzon G., Marinelli C., D’Incà R. (2020). Microbiota Changes Induced by Microencapsulated Sodium Butyrate in Patients with Inflammatory Bowel Disease. Neurogastroenterol. Motil..

[40] Dahal R.H., Kim S., Kim Y.K., Kim E.S., Kim J. (2023). Insight into Gut Dysbiosis of Patients with Inflammatory Bowel Disease and Ischemic Colitis. Front. Microbiol..

[41] Sasaki K., Inoue J., Sasaki D., Hoshi N., Shirai T., Fukuda I., Azuma T., Kondo A., Osawa R. (2019). Construction of a Model Culture System of Human Colonic Microbiota to Detect Decreased Lachnospiraceae Abundance and Butyrogenesis in the Feces of Ulcerative Colitis Patients. Biotechnol. J..

[42] Liu S., Zhao W., Lan P., Mou X. (2021). The Microbiome in Inflammatory Bowel Diseases: From Pathogenesis to Therapy. Protein Cell.

[43] Chang Q., Luan Y., Sun F. (2011). Variance Adjusted Weighted UniFrac: A Powerful Beta Diversity Measure for Comparing Communities Based on Phylogeny. BMC Bioinform..

[44] Parada Venegas D., De la Fuente M.K., Landskron G., González M.J., Quera R., Dijkstra G., Harmsen H.J.M., Faber K.N., Hermoso M.A. (2019). Short Chain Fatty Acids (SCFAs)-Mediated Gut Epithelial and Immune Regulation and Its Relevance for Inflammatory Bowel Diseases. Front. Immunol..

[45] Peace C.G., O’Neill L.A. (2022). The Role of Itaconate in Host Defense and Inflammation. J. Clin. Investig..

[46] Clooney A.G., Eckenberger J., Laserna-Mendieta E., Sexton K.A., Bernstein M.T., Vagianos K., Sargent M., Ryan F.J., Moran C., Sheehan D. (2021). Ranking Microbiome Variance in Inflammatory Bowel Disease: A Large Longitudinal Intercontinental Study. Gut.

[47] Abdel-Rahman L.I.H., Morgan X.C. (2023). Searching for a Consensus Among Inflammatory Bowel Disease Studies: A Systematic Meta-Analysis. Inflamm. Bowel Dis..

[48] Nie K., Ma K., Luo W., Shen Z., Yang Z., Xiao M., Tong T., Yang Y., Wang X. (2021). Roseburia Intestinalis: A Beneficial Gut Organism from the Discoveries in Genus and Species. Front. Cell. Infect. Microbiol..

[49] Luo W., Shen Z., Deng M., Li X., Tan B., Xiao M., Wu S., Yang Z., Zhu C., Tian L. (2019). Roseburia Intestinalis Supernatant Ameliorates Colitis Induced in Mice by Regulating the Immune Response. Mol. Med. Rep..

[50] Tilg H., Danese S. (2014). Roseburia Hominis: A Novel Guilty Player in Ulcerative Colitis Pathogenesis?. Gut.

[51] Alexeev E.E., Lanis J.M., Kao D.J., Campbell E.L., Kelly C.J., Battista K.D., Gerich M.E., Jenkins B.R., Walk S.T., Kominsky D.J. (2018). Microbiota-Derived Indole Metabolites Promote Human and Murine Intestinal Homeostasis through Regulation of Interleukin-10 Receptor. Am. J. Pathol..

[52] Wu H., Zhang M., Li W., Zhu S., Zhang D. (2020). Stachydrine Attenuates IL-1β-Induced Inflammatory Response in Osteoarthritis Chondrocytes through the NF-ΚB Signaling Pathway. Chem. Biol. Interact..

[53] Gao Y., Zhou B., Zhang H., Chen L., Wang X., Chen H., Zhou L. (2022). L-Ergothioneine Exhibits Protective Effects against Dextran Sulfate Sodium-Induced Colitis in Mice. ACS Omega.

[54] Gründemann D., Harlfinger S., Golz S., Geerts A., Lazar A., Berkels R., Jung N., Rubbert A., Schömig E. (2005). Discovery of the Ergothioneine Transporter. Proc. Natl. Acad. Sci. USA.

[55] Bryan P.-F., Karla C., Edgar Alejandro M.-T., Sara Elva E.-P., Gemma F., Luz C. (2016). Sphingolipids as Mediators in the Crosstalk between Microbiota and Intestinal Cells: Implications for Inflammatory Bowel Disease. Mediat. Inflamm..

[56] Hu D., Zhang D., Zheng S., Guo M., Lin X., Jiang Y. (2016). Association of Ulcerative Colitis with FUT2 and FUT3 Polymorphisms in Patients from Southeast China. PLoS ONE.

[57] Kappler K., Lasanajak Y., Smith D.F., Opitz L., Hennet T. (2020). Increased Antibody Response to Fucosylated Oligosaccharides and Fucose-Carrying Bacteroides Species in Crohn’s Disease. Front. Microbiol..

[58] Vermeulen N., Vermeire S., Arijs I., Michiels G., Ballet V., Derua R., Waelkens E., Van Lommel L., Schuit F., Rutgeerts P. (2011). Seroreactivity against Glycolytic Enzymes in Inflammatory Bowel Disease. Inflamm. Bowel Dis..

[59] Hryhorowicz S., Kaczmarek-Ryś M., Zielińska A., Scott R.J., Słomski R., Pławski A. (2021). Endocannabinoid System as a Promising Therapeutic Target in Inflammatory Bowel Disease—A Systematic Review. Front. Immunol..

[60] Maki J.J., Nielsen D.W., Looft T. (2020). Complete Genome Sequence and Annotation for Turicibacter Sanguinis MOL361T (DSM 14220). Microbiol Resour Announc.

[61] Lim S., Rhee M.-S., Chang D.-H., Kim B.-C. (2016). Whole-Genome Sequence of Erysipelothrix Larvae LV19T (=KCTC 33523T), a Useful Strain for Arsenic Detoxification, from the Larval Gut of the Rhinoceros Beetle, Trypoxylus Dichotomus. J. Biotechnol..

[62] Chen L.-M., Bao C.-H., Wu Y., Liang S.-H., Wang D., Wu L.-Y., Huang Y., Liu H.-R., Wu H.-G. (2021). Tryptophan-Kynurenine Metabolism: A Link between the Gut and Brain for Depression in Inflammatory Bowel Disease. J. Neuroinflamm..

[63] Rustgi S.D., Kayal M., Shah S.C. (2020). Sex-Based Differences in Inflammatory Bowel Diseases: A Review. Therap. Adv. Gastroenterol..

[64] Sochal M., Ditmer M., Binienda A., Gabryelska A., Białasiewicz P., Talar-Wojnarowska R., Fichna J., Małecka-Wojciesko E. (2023). Relation between Selected Sleep Parameters, Depression, Anti-Tumor Necrosis Factor Therapy, and the Brain-Derived Neurotrophic Factor Pathway in Inflammatory Bowel Disease. Metabolites.

[65] Jo J.-K., Seo S.-H., Park S.-E., Kim H.-W., Kim E.-J., Kim J.-S., Pyo J.-Y., Cho K.-M., Kwon S.-J., Park D.-H. (2021). Gut Microbiome and Metabolome Profiles Associated with High-Fat Diet in Mice. Metabolites.

