# Supplementary material for: Mucosal Metabolomic Signatures in Chronic Colitis: Novel Insights into the Pathophysiology of Inflammatory Bowel Disease
Source: Metabolites. 2023 Jul 23;13(7):873. doi: 10.3390/metabo13070873 (PMC10386370; doi:10.3390/metabo13070873)
Supplement: Supplementary file 1 [file metabolites-13-00873-s001.zip › Metabolites Journal Supp.pptx]

## Slide 1
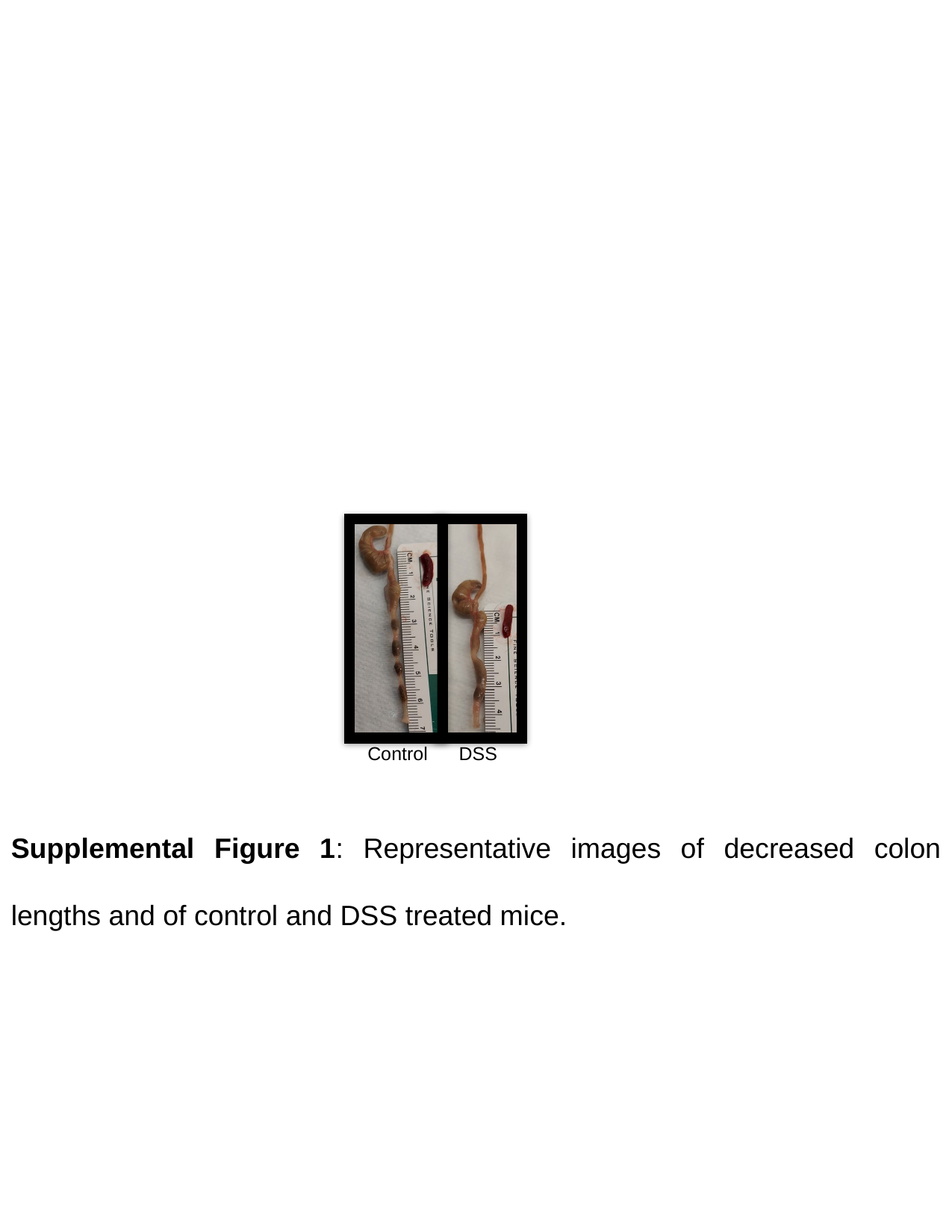

Control DSS
Supplemental Figure 1: Representative images of decreased colon lengths and of control and DSS treated mice.

## Slide 2
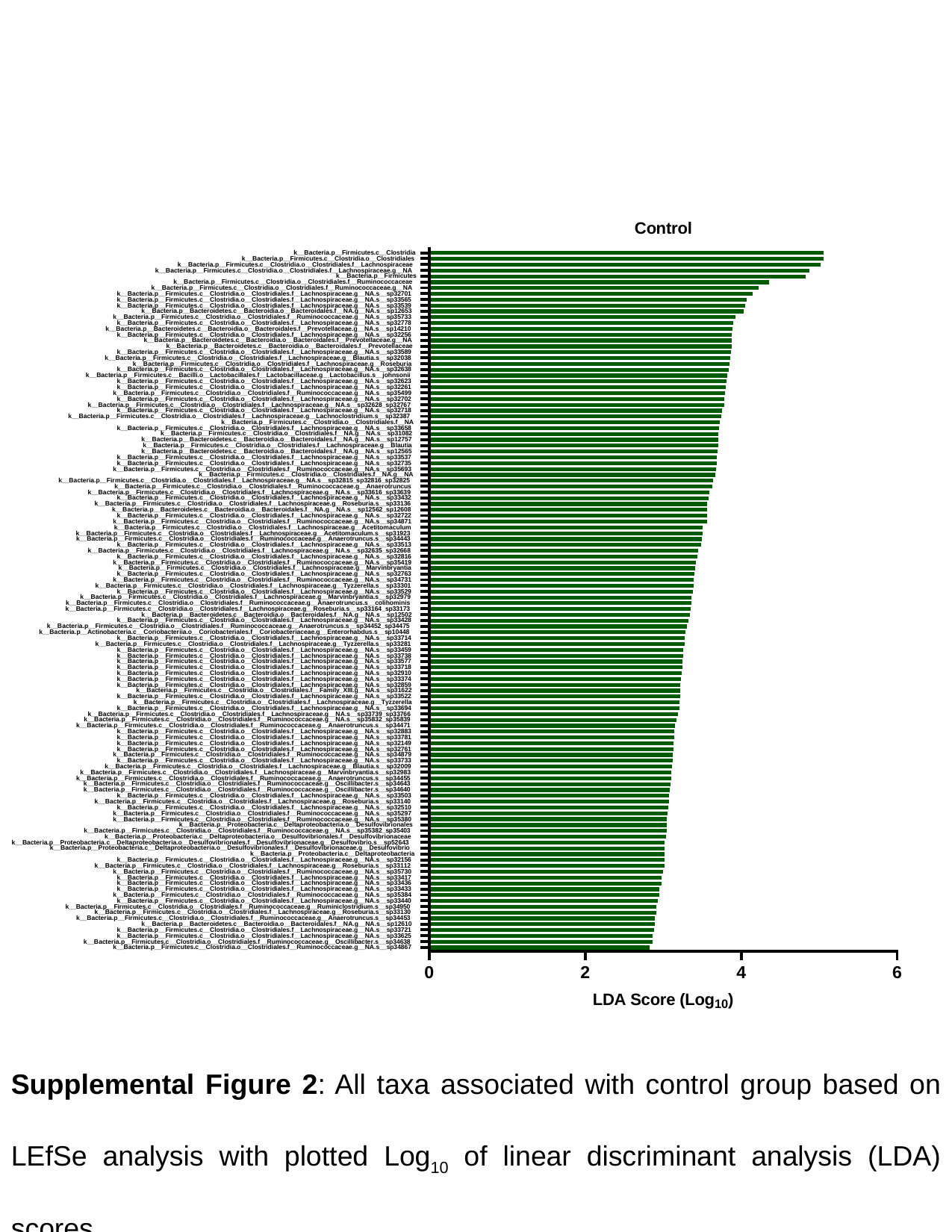

Supplemental Figure 2: All taxa associated with control group based on LEfSe analysis with plotted Log10 of linear discriminant analysis (LDA) scores.

## Slide 3
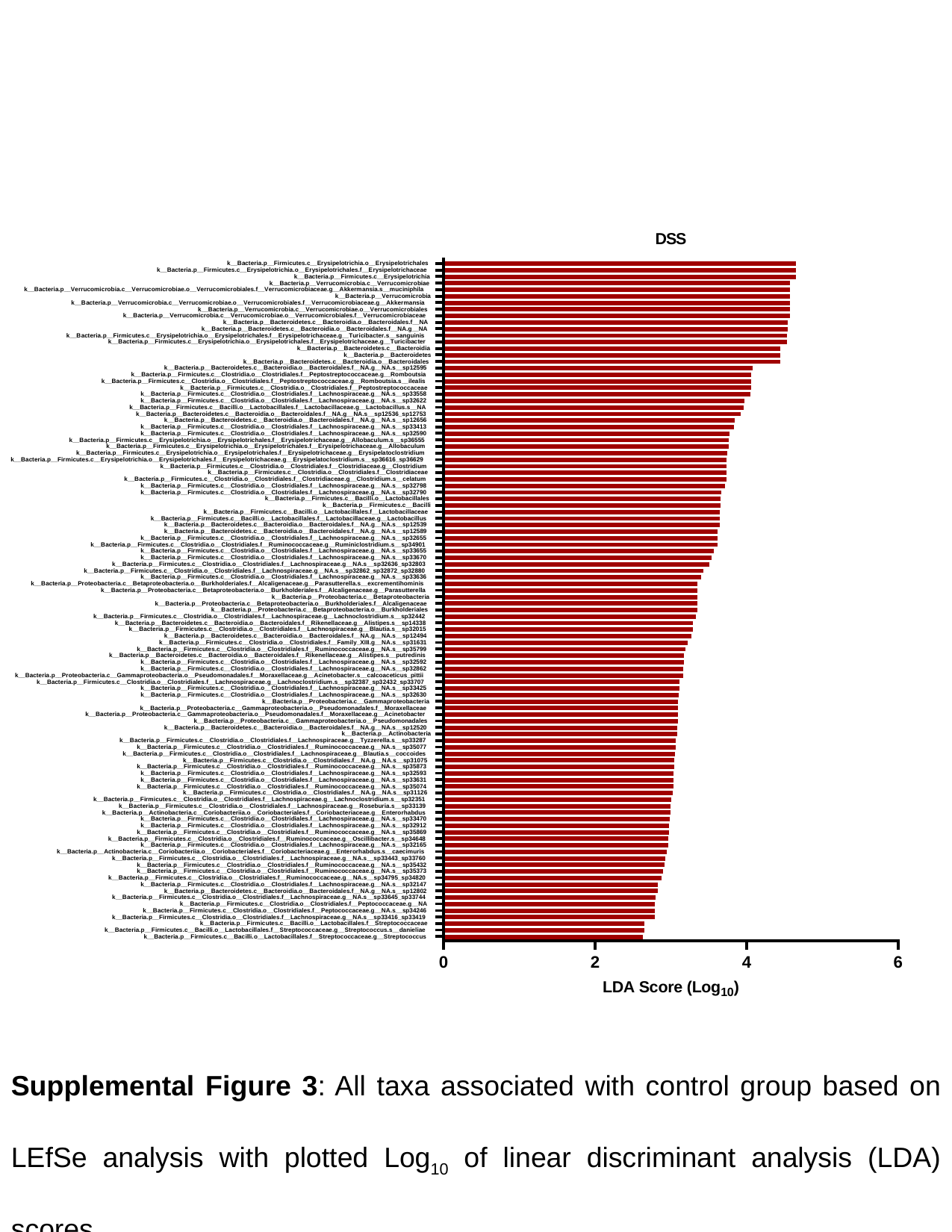

Supplemental Figure 3: All taxa associated with control group based on LEfSe analysis with plotted Log10 of linear discriminant analysis (LDA) scores.

## Slide 4
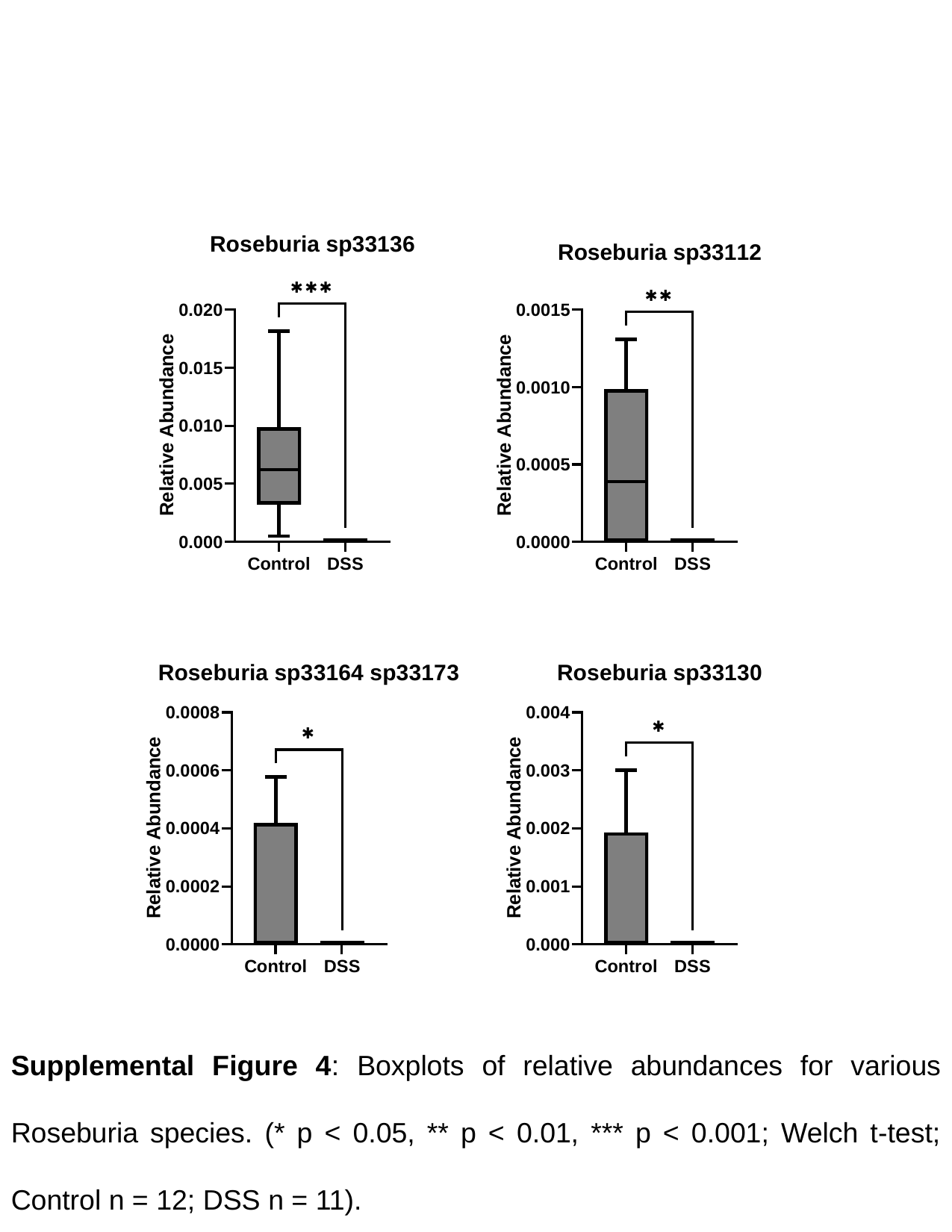

Supplemental Figure 4: Boxplots of relative abundances for various Roseburia species. (* p < 0.05, ** p < 0.01, *** p < 0.001; Welch t-test; Control n = 12; DSS n = 11).

## Slide 5
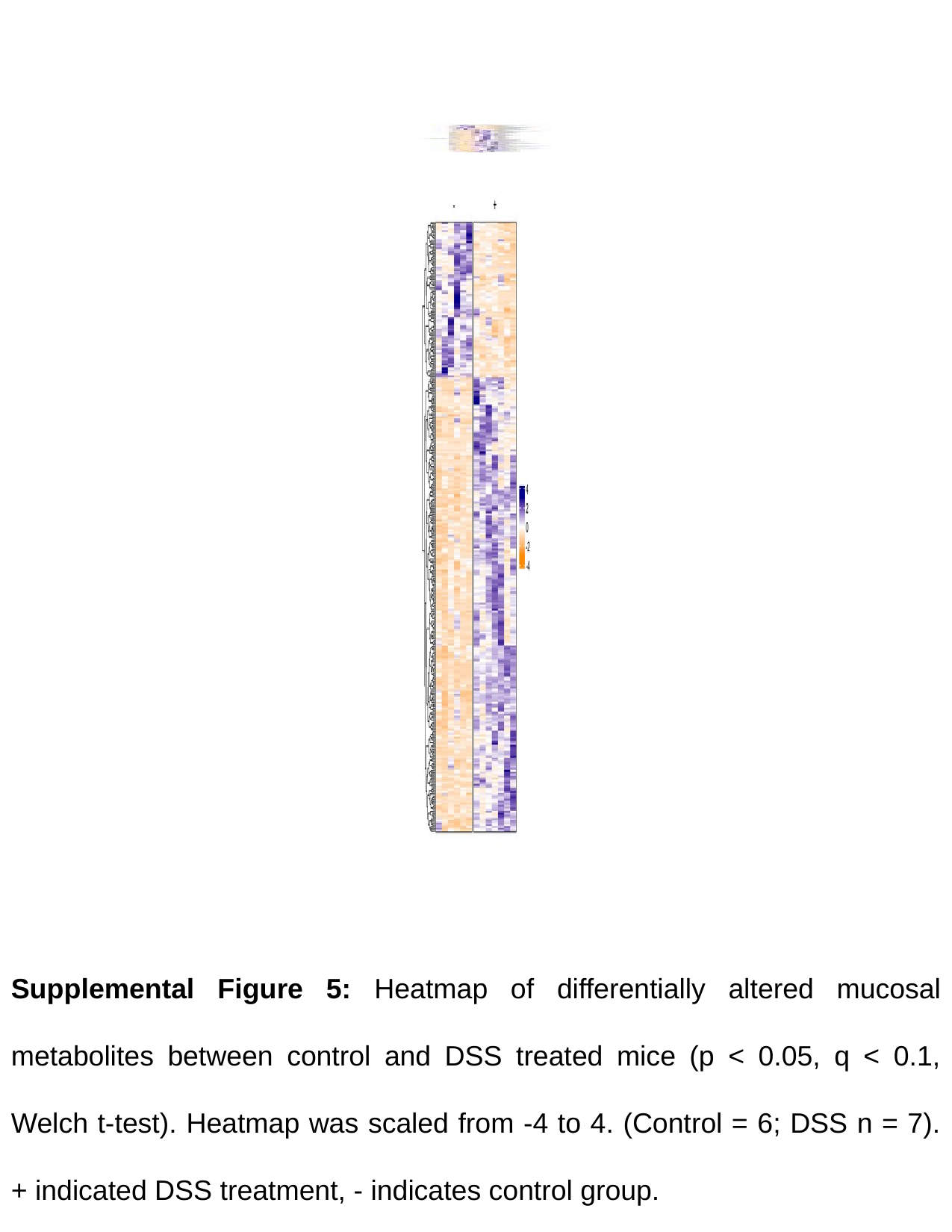

Supplemental Figure 5: Heatmap of differentially altered mucosal metabolites between control and DSS treated mice (p < 0.05, q < 0.1, Welch t-test). Heatmap was scaled from -4 to 4. (Control = 6; DSS n = 7). + indicated DSS treatment, - indicates control group.

## Slide 6
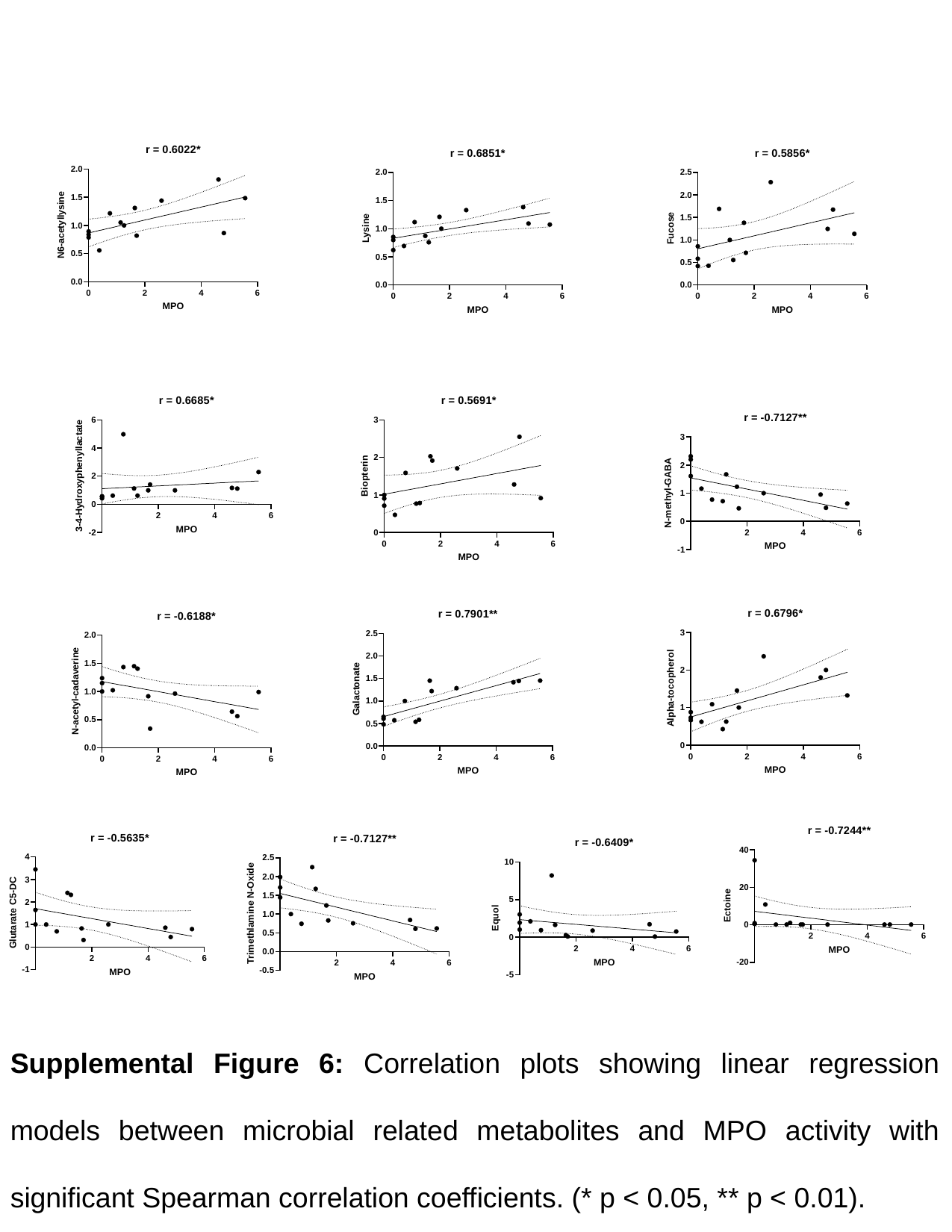

Supplemental Figure 6: Correlation plots showing linear regression models between microbial related metabolites and MPO activity with significant Spearman correlation coefficients. (* p < 0.05, ** p < 0.01).

## Slide 7
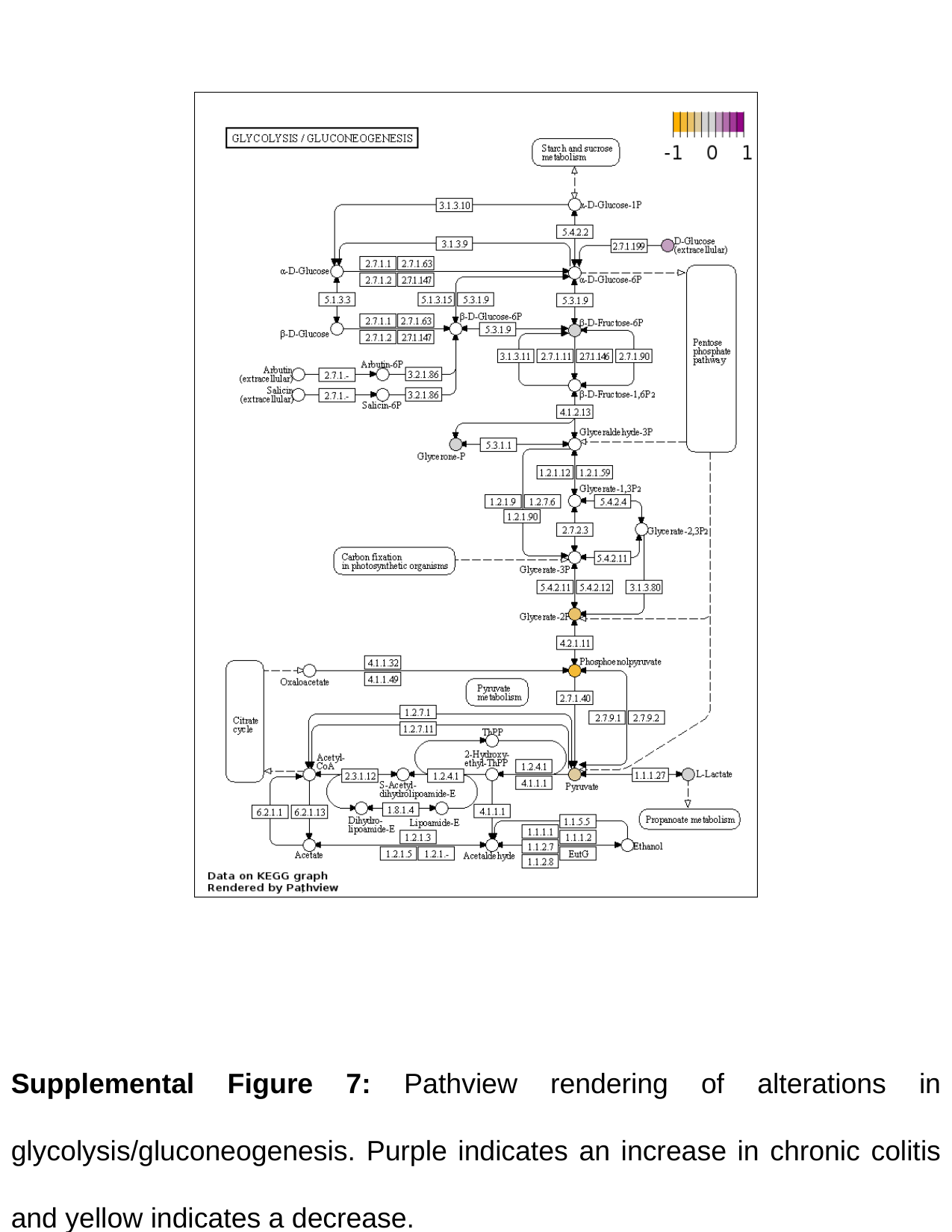

Supplemental Figure 7: Pathview rendering of alterations in glycolysis/gluconeogenesis. Purple indicates an increase in chronic colitis and yellow indicates a decrease.

## Slide 8
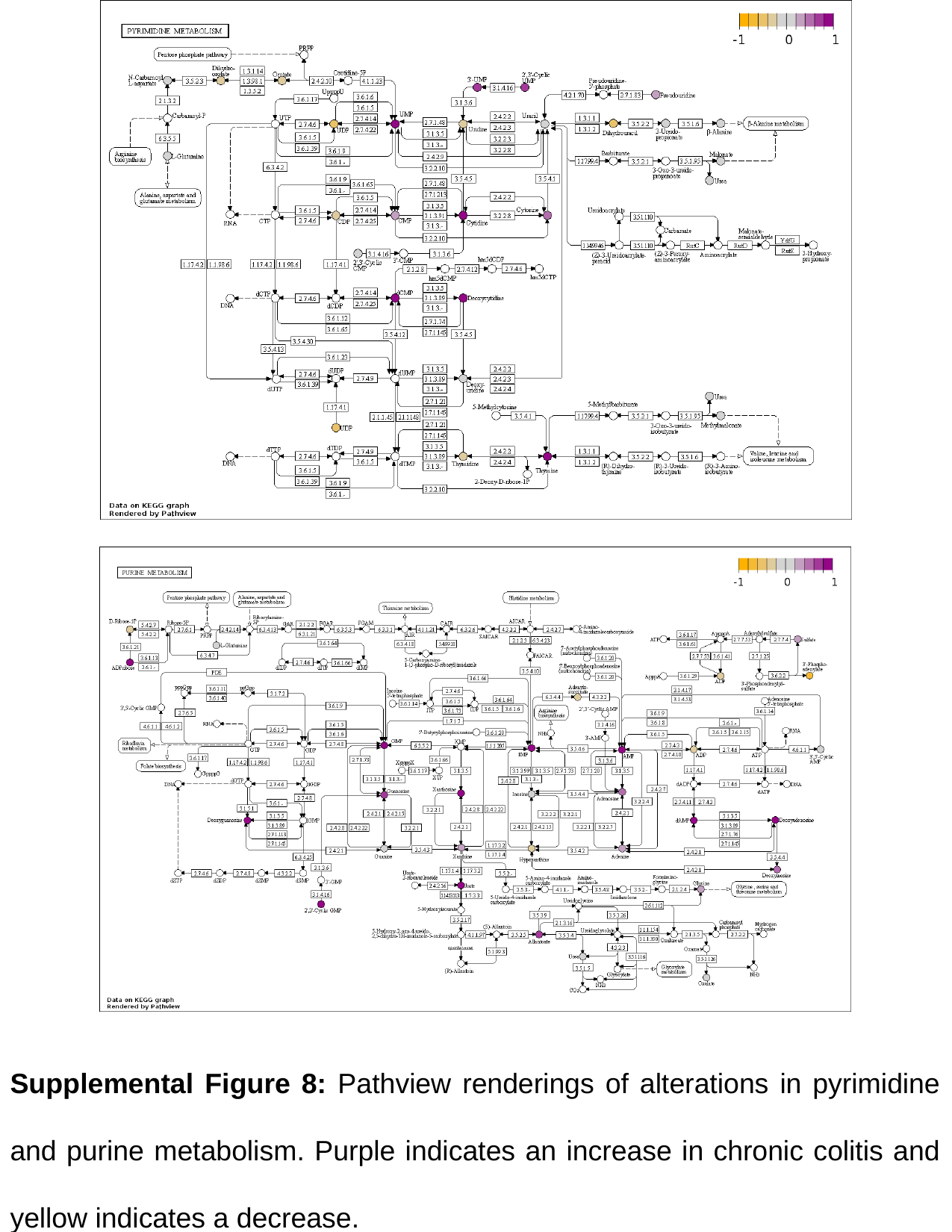

Supplemental Figure 8: Pathview renderings of alterations in pyrimidine and purine metabolism. Purple indicates an increase in chronic colitis and yellow indicates a decrease.

## Slide 9
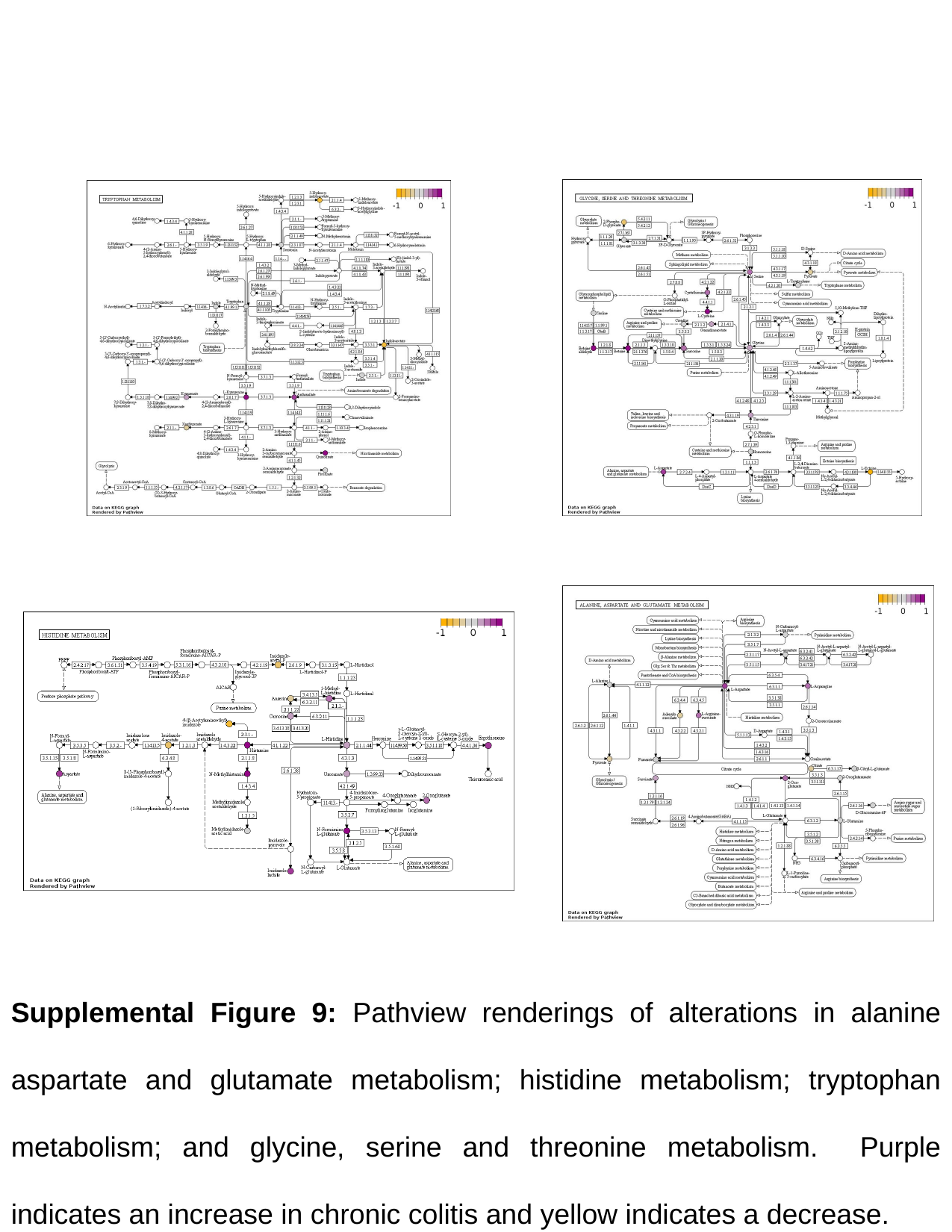

Supplemental Figure 9: Pathview renderings of alterations in alanine aspartate and glutamate metabolism; histidine metabolism; tryptophan metabolism; and glycine, serine and threonine metabolism. Purple indicates an increase in chronic colitis and yellow indicates a decrease.

## Slide 10
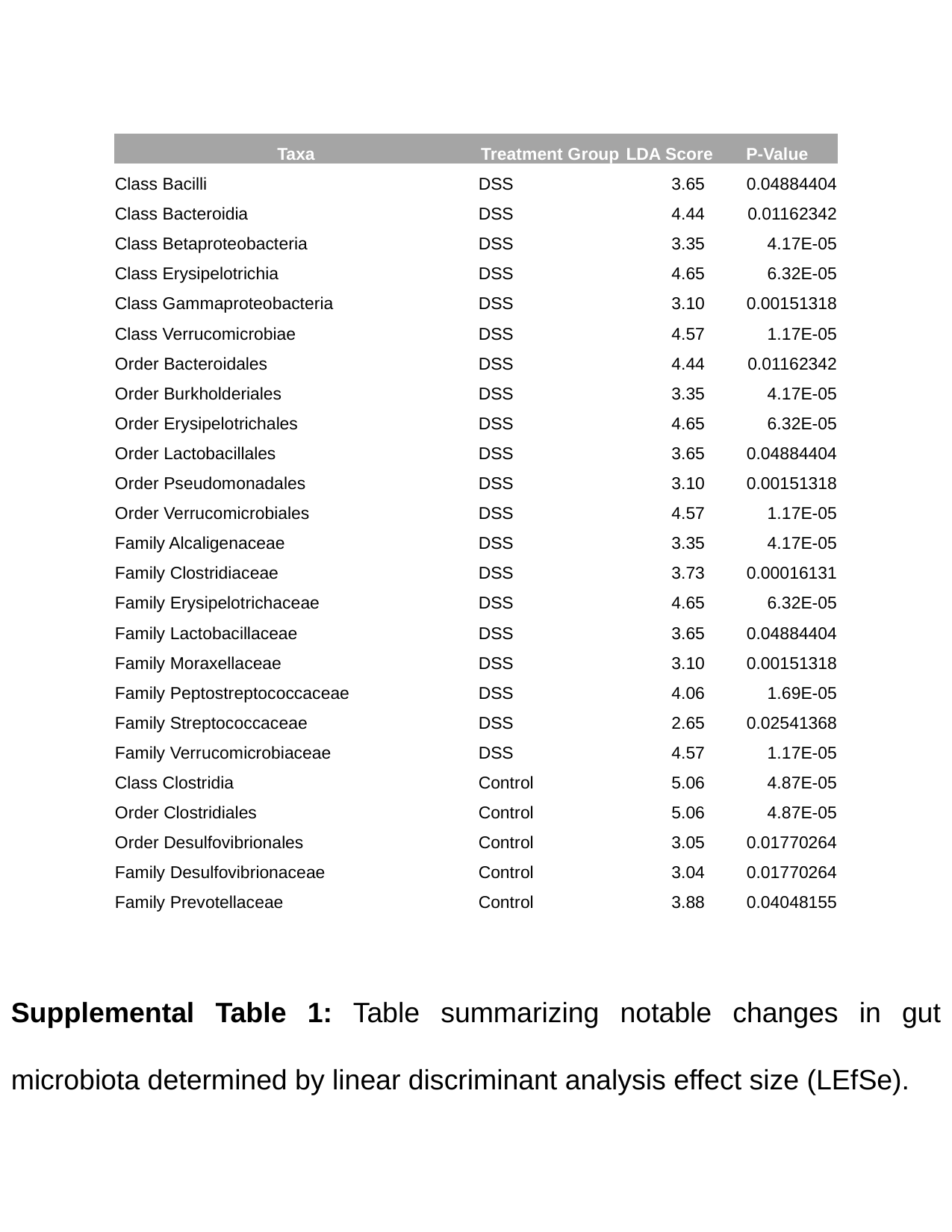

| Taxa | Treatment Group | LDA Score | P-Value |
| --- | --- | --- | --- |
| Class Bacilli | DSS | 3.65 | 0.04884404 |
| Class Bacteroidia | DSS | 4.44 | 0.01162342 |
| Class Betaproteobacteria | DSS | 3.35 | 4.17E-05 |
| Class Erysipelotrichia | DSS | 4.65 | 6.32E-05 |
| Class Gammaproteobacteria | DSS | 3.10 | 0.00151318 |
| Class Verrucomicrobiae | DSS | 4.57 | 1.17E-05 |
| Order Bacteroidales | DSS | 4.44 | 0.01162342 |
| Order Burkholderiales | DSS | 3.35 | 4.17E-05 |
| Order Erysipelotrichales | DSS | 4.65 | 6.32E-05 |
| Order Lactobacillales | DSS | 3.65 | 0.04884404 |
| Order Pseudomonadales | DSS | 3.10 | 0.00151318 |
| Order Verrucomicrobiales | DSS | 4.57 | 1.17E-05 |
| Family Alcaligenaceae | DSS | 3.35 | 4.17E-05 |
| Family Clostridiaceae | DSS | 3.73 | 0.00016131 |
| Family Erysipelotrichaceae | DSS | 4.65 | 6.32E-05 |
| Family Lactobacillaceae | DSS | 3.65 | 0.04884404 |
| Family Moraxellaceae | DSS | 3.10 | 0.00151318 |
| Family Peptostreptococcaceae | DSS | 4.06 | 1.69E-05 |
| Family Streptococcaceae | DSS | 2.65 | 0.02541368 |
| Family Verrucomicrobiaceae | DSS | 4.57 | 1.17E-05 |
| Class Clostridia | Control | 5.06 | 4.87E-05 |
| Order Clostridiales | Control | 5.06 | 4.87E-05 |
| Order Desulfovibrionales | Control | 3.05 | 0.01770264 |
| Family Desulfovibrionaceae | Control | 3.04 | 0.01770264 |
| Family Prevotellaceae | Control | 3.88 | 0.04048155 |
Supplemental Table 1: Table summarizing notable changes in gut microbiota determined by linear discriminant analysis effect size (LEfSe).

## Slide 11
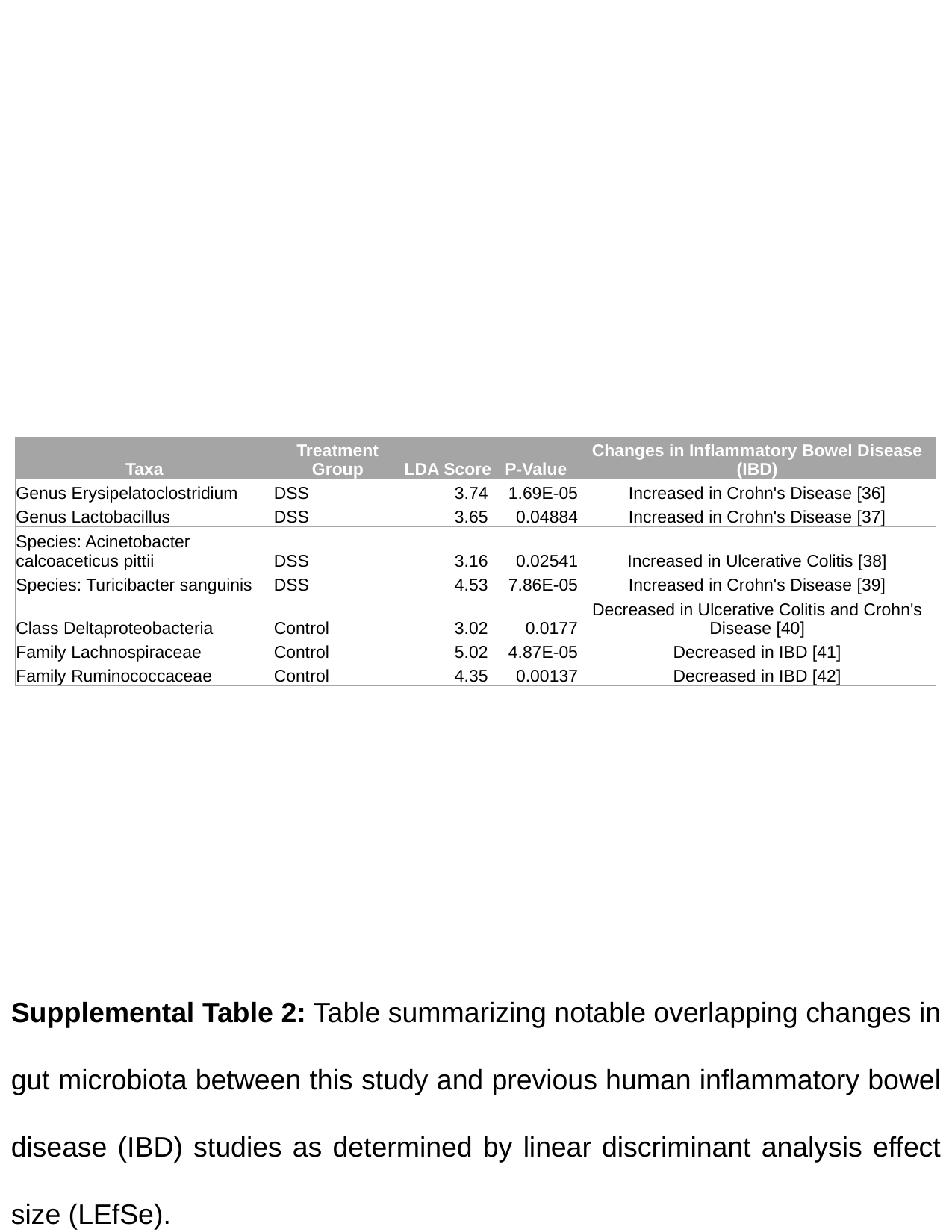

| Taxa | Treatment Group | LDA Score | P-Value | Changes in Inflammatory Bowel Disease (IBD) |
| --- | --- | --- | --- | --- |
| Genus Erysipelatoclostridium | DSS | 3.74 | 1.69E-05 | Increased in Crohn's Disease [36] |
| Genus Lactobacillus | DSS | 3.65 | 0.04884 | Increased in Crohn's Disease [37] |
| Species: Acinetobacter calcoaceticus pittii | DSS | 3.16 | 0.02541 | Increased in Ulcerative Colitis [38] |
| Species: Turicibacter sanguinis | DSS | 4.53 | 7.86E-05 | Increased in Crohn's Disease [39] |
| Class Deltaproteobacteria | Control | 3.02 | 0.0177 | Decreased in Ulcerative Colitis and Crohn's Disease [40] |
| Family Lachnospiraceae | Control | 5.02 | 4.87E-05 | Decreased in IBD [41] |
| Family Ruminococcaceae | Control | 4.35 | 0.00137 | Decreased in IBD [42] |
Supplemental Table 2: Table summarizing notable overlapping changes in gut microbiota between this study and previous human inflammatory bowel disease (IBD) studies as determined by linear discriminant analysis effect size (LEfSe).
